# Discovery of midgut genes for the RNA interference control of corn rootworm

**DOI:** 10.1038/srep30542

**Published:** 2016-07-28

**Authors:** Xu Hu, Nina M. Richtman, Jian-Zhou Zhao, Keith E. Duncan, Xiping Niu, Lisa A. Procyk, Meghan A. Oneal, Bliss M. Kernodle, Joseph P. Steimel, Virginia C. Crane, Gary Sandahl, Julie L. Ritland, Richard J. Howard, James K. Presnail, Albert L. Lu, Gusui Wu

**Affiliations:** 1 DuPont Pioneer, Johnston, IA, USA; 2 DuPont Pioneer, Wilmington, DE, USA

## Abstract

RNA interference (RNAi) is a promising new technology for corn rootworm control. This paper presents the discovery of new gene targets - *dvssj1* and *dvssj2*, in western corn rootworm (WCR). *Dvssj1* and *dvssj2* are orthologs of the *Drosophila* genes snakeskin (*ssk*) and *mesh*, respectively. These genes encode membrane proteins associated with smooth septate junctions (SSJ) which are required for intestinal barrier function. Based on bioinformatics analysis, *dvssj1* appears to be an arthropod-specific gene. Diet based insect feeding assays using double-stranded RNA (dsRNA) targeting *dvssj1* and *dvssj2* demonstrate targeted mRNA suppression, larval growth inhibition, and mortality. In RNAi treated WCR, injury to the midgut was manifested by “blebbing” of the midgut epithelium into the gut lumen. Ultrastructural examination of midgut epithelial cells revealed apoptosis and regenerative activities. Transgenic plants expressing dsRNA targeting *dvssj1* show insecticidal activity and significant plant protection from WCR damage. The data indicate that *dvssj1* and *dvssj2* are effective gene targets for the control of WCR using RNAi technology, by apparent suppression of production of their respective smooth septate junction membrane proteins located within the intestinal lining, leading to growth inhibition and mortality.

The western corn rootworm (WCR), *Diabrotica virgifera virgifera* LeConte (Coleoptera: Chrysomelidae), is one of the most devastating pests in maize that can cause economic losses exceeding $1 billion annually in the U.S.A.^1^. WCR has traditionally been managed through crop rotation and broad-spectrum soil insecticides^2^. For over a decade, rootworm management has mainly focused on transgenic corn hybrids expressing *Bacillus thuringiensis* (*Bt*) toxins^3^. Currently, four *Bt* toxins (Cry3Bb1, mCry3A, eCry3.1Ab and Cry34/35Ab1), are used commercially for the control of WCR and are expressed in corn hybrids either singly or as pyramids^4^. Recent reports of emerging field insect resistance to both mCry3A and Cry3Bb1 demonstrate the need for effective insect resistance management strategies and discovery of new traits^5^,^6^.

RNA interference (RNAi) is a naturally occurring mechanism that regulates gene expression and anti-viral defense in most plants and animals^7^ and has become an important tool for reverse functional genomics and applications in biomedicine and agriculture^8^,^9^. Demonstration of RNA interference following delivery of dsRNA *via* oral ingestion was first shown in *Caenorhabditis elegans*^10^ and has since been documented extensively in insects including WCR^11^,^12^,^13^. Once dsRNA is taken up by cells, dicer RNase III type enzymes bind and digest cytoplasmic dsRNAs into small interfering RNAs (siRNAs) associated with an RNA-induced silencing complex (RISC). The argonaute proteins of RISC cleave the target mRNA strand complementary to their bound siRNA, which determines the specificity of the RNAi activities through precise base-pairing recognition of their complementary target RNAs^14^. Many genes have been reported to be potential targets in WCR following the provision of dsRNA in diet bioassay^13^,^15^,^16^ and demonstrate that WCR is sensitive to orally delivered dsRNA, providing a new management approach for this important pest^17^,^18^.

Pest control *via* RNA interference has been demonstrated *in planta* by expressing dsRNA targeted toward the “housekeeping” genes *α-tubulin, V-ATPase A*^13^, and C subunit^19^ or genes involved in cellular pathways such as *snf7*^20^. V-ATPases are highly conserved multisubunit enzymes that function to acidify intracellular organelles by pumping protons across plasma membranes in exchange for energy^21^. *Snf7* encodes a vacuolar sorting protein involved in intracellular protein trafficking^22^. Finding new classes of WCR RNAi targets (“modes of action”) is important for effective management of WCR in the future.

The insect midgut plays a critical role in the regulation of important physiological functions such as digestion, metabolism, immune response, electrolyte homoeostasis, osmotic pressure, and circulation^23^,^24^. Impairment of one or more of these functions provides a potential basis for new pest management approaches utilizing RNAi. The midgut epithelial cells of most invertebrate species possess specialized cell–cell junctions, known as septate junctions (SJ)^25^,^26^, that display a characteristic electron-dense ladder-like structure of 10–20 nm width^27^. SJs typically form circumferential belts around the apicolateral regions of epithelial cells and control the paracellular pathway^26^. SJs are subdivided into several morphological types that vary among different animal phyla and different types of SJ have been described in different epithelia within an individual in several phyla^25^. Molecular and genetic analyses of SJs of invertebrate species have only been performed in *Drosophila*^28^,^29^, where two types of SJ are present: pleated SJ (PSJ) and smooth SJ (SSJ), in ectodermally and endodermally derived epithelia, respectively^28^. More than 20 PSJ-related proteins have been identified and characterized^28^. These include transmembrane [e.g. Fasciclin II (FasII), Fasciclin III (FasIII)] and cytoplasmic proteins [e.g. Coracle (Cora), Discs large (Dlg), Lethal (2) giant larvae (Lgl)] localizing at PSJs. In contrast, only two SSJ-specific proteins encoded by *Drosophila* genes snakeskin (*ssk*) and *mesh* have been reported^30^,^31^. SSK and MESH form a complex and the two proteins are mutually interdependent for their correct localization^31^. Several PSJ components, including Dlg, Lgl, Cora and FasIII, have been confirmed to localize to the SSJs. In *ssk*-and *mesh*-deficient midguts, Lgl, Cora and FasIII are mislocalized but Dlg is not^31^. The functions of these PSJ proteins in SSJs remain uncertain since Dlg, Lgl, Cora and FasIII are not required for the SSJ localization of MESH and SSK, and are dispensable for SSJ formation^28^. The molecular composition of SSJs is different from that of PSJs^28^. Genetic studies in *Drosophila* have shown that fluorescent-labeled dextrans (10 kDa) are unable to pass between midgut epithelial cells in wild-type flies but are able to penetrate the paracellular route in mutants defective for smooth septate formation^28^. The *ssk*-RNAi and *ssk-deletion* mutants were lethal at late stage 17 of *Drosophila* embryo. *Ssk* and *mesh* are required for *Drosophila* development, SSJ formation and midgut paracellular barrier function^30^,^31^.

Here we present the discovery of two WCR midgut genes that can potentially serve as effective insecticidal targets using RNA interference technology. *Dvssj1* appears to be an arthropod-specific gene that is not found in vertebrates or plants. Insect diet-based assays demonstrated WCR gene target specific mRNA suppression, larval growth inhibition, and mortality. In addition, transgenic maize expressing dsRNA to one of these gene targets (*dvssj1*) showed a significant reduction in root damage by WCR.

## Results

### Identification of WCR gene targets

A WCR diet bioassay system for dsRNA-based random screening was developed to identify new and highly active RNAi targets for RNAi-mediated pest control. Double-stranded RNA was produced by *in vitro* transcription (IVT) and incorporated into WCR diet at a final concentration of 50 ng μl^−1^ in a 96 well plate format. Insects were scored for mortality and stunting after 7 days and an average primary score was assigned based on 8 observations (replicates) for each dsRNA target. Active target genes (scores ≥ 2) were confirmed and further characterized. Two midgut genes, *dvssj1* and *dvssj2* (Table 1) were identified among a cohort of 35 WCR RNAi active targets (Supplementary Table 1a).

A set of dsRNA’s targeting *dvssj1* and *dvssj2,* and representing different subfragments of the respective full length sequences were further evaluated in WCR feeding assays to identify fragments with improved efficacy. Fragments with a score ≥2 were selected to determine 50% lethal concentration (LC_50_) and 50% inhibition concentration (IC_50_) values (Table 1). *Dvssj1* frag1 was the most active dsRNA possessing an LC_50_ of 0.041 ng μl^−1^. In contrast, *dvssj2* fragments were about 2 to 7-fold less active with a range of LC_50_ from 0.089 to 0.286 ng μl^−1^.

Time to 50% lethality (LT_50_) was measured for *dvssj1* and *dvssj2* and other active fragments of *dvpat3*, *dvprotb* and *dvrps10* (see Supplementary Table 2). *Dvssj1* and *dvssj2* (Table 2) had significantly shorter LT_50_ than the other active targets. The LT_50_ (5 ng μl^−1^) for *dvssj1* and *dvssj2* was 6.6 and 7.1 days, respectively, compared to LT_50_ > 8 days for the other active fragments in the assay. Overall, *dvssj1* had the shortest LT_50_ of the RNAi actives tested (Table 2).

### *SSJ* targets are midgut genes

**Figure 1:**
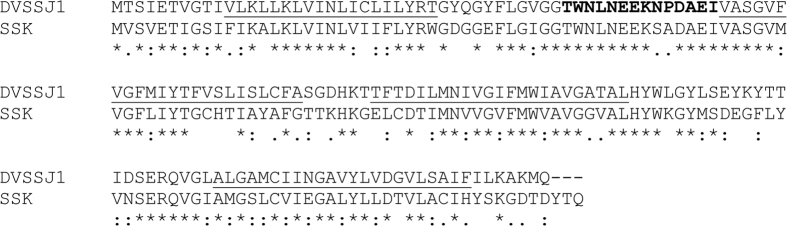
Protein alignments of DVSSJ1 and *Drosophila* SSK. The transmembrane domains with hydrophobic residues are indicated by underline predicted by the SOSUI algorithm^50^. The bold letters indicate the amino acid sequence (TWNLNEEKNPDAEIC) used for monoclonal antibody production. This alignment was derived using CLUSTAL W with default parameters^48^. * (asterisk) represents identical amino acid residues shared between DVSSJ1 and fly-SSK, : (colon) conservation between two amino acid residues of strongly similar properties and. (period) indicates conservation between two amino acid residues of weakly similar properties.

**Figure 2:**
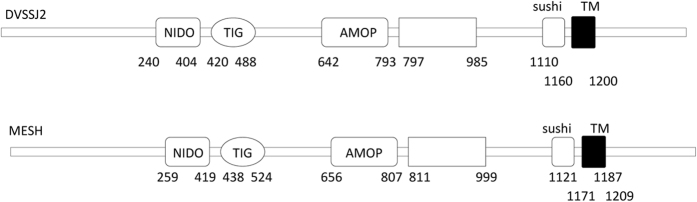
Schematic representation of DVSSJ2 and *Drosophila* MESH domain structure showing conservation of domains. DVSSJ2 has a single-pass transmembrane (TM) domain, a large extracellular region containing a NIDO (Nidogen-like) domain, aTIG (Transcription factor ImmunoGlobin) domain, an AMOP domain, a VWD (von Willebrand factor type D) domain, and SUSHI repeats predicted by the Pfam protein families database^49^.

*Dvssj1* and *dvssj2* were named based on their homology to previously characterized smooth septate junction (SSJ) genes from *Drosphilia, ssk*^30^ and *mesh*^31^, respectively. DVSSJ1 and DVSSJ2 are 54.9% and 51.3% identical at the amino acid sequence level to SSK (Fig. 1) and MESH (Supplemental Fig. 1), respectively. Both *mesh* and *ssk* are required for SSJ formation in the *Drosophila* midgut^30^,^31^. *Dvssj1* encodes a 160 amino acid protein with a predicted molecular weight of 17.6 kDa and four predicted membrane-spanning domains (Fig. 1). *Dvssj2* has a predicted protein sequence of 1357 amino acids and a MW of 155.9 kDa. The primary structure of DVSSJ2 is similar to MESH in *Drosophila*^31^ and contains a single-pass transmembrane (TM) domain, a large extracellular region containing a NIDO (Nidogen-like domain), a TIG (Transcription factor ImmunoGlobin) domain, AMOP^32^ domain, a VWD (von Willebrand factor type D) domain, and SUSHI repeats (Fig. 2).

### Target expression and suppression by dsRNA feeding

**Figure 3:**
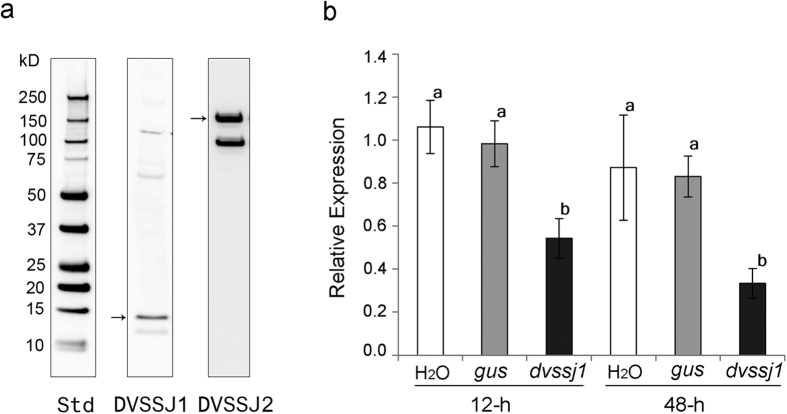
Expression analyses of DVSSJ proteins and *dvssj1* mRNA. (**a**) Western blot detection of DVSSJ1 and DVSSJ2 from 3^rd^ instar WCR dissected gut tissues. Loaded samples represent the equivalent of 1 WCR midgut. Detection of DVSSJ1 is by anti-DVSSJ1 monoclonal peptide antibody (Peptides sequence: TWNLNEEKNPDAEI 41-53 a.a.). DVSSJ2 was detected by Hybridoma Supernatant from peptide antibody production. (Peptide Sequence: MTSDTAPPDTDQRG 108-121 a.a.). The DVSSJ1 and DVSSJ2 detectable protein sizes are compared by Precision Plus Protein Western Standard (Std, Bio-Rad) ranging from 10–250 kDa (Supplementary Fig. 2). (**b**) Relative gene expression of *dvssj1* over time by dsRNA treatment. qRT-PCR was used to examine gene expression of *dvssj1*. The expression represented relative expression for time points 12 and 48 hrs for treatments of double stranded RNA for *dvssj1* and *gus*. H_2_0 was used as a control treatment. Relative expression analysis was based on *dvssj1* expression, after being normalized by reference gene *dvrps10* expression, and then compared to *dvssj1* expression in H_2_0 control at each time point. Each value was shown as values ± S.E.M. of individual insects. Letter differences represent treatments that are significantly different from each other (P-value < 0.05) determined by one-way analysis of variance (ANOVA) followed by Tukey’s post-test.

*Ssk* and *mesh* in *Drosophila* are specifically expressed in endodermally derived epithelia, including the midgut, gastric caeca, the outer epithelial layer of the proventriculus, and the Malpighian tubules^30^,^31^. Proteins of both *dvssj1* and *dvssj2* were detected in midgut homogenates extracted from 3^rd^ instar WCR with expected MWs (arrows) of 17.6 and 155.9 kDa, respectively (Fig. 3a and Supplementary Fig. 2).

**Figure 4:**
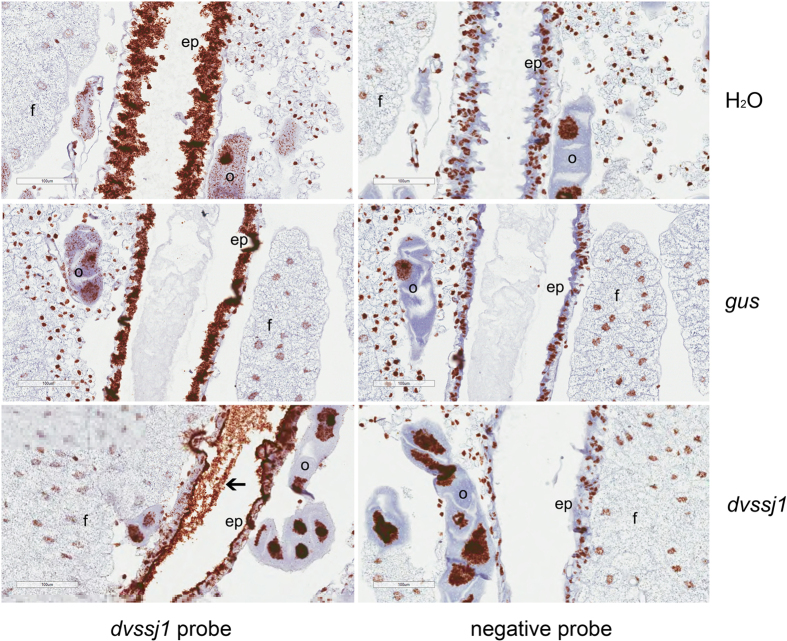
Visualization of *dvssj1* mRNA expression in WCR 3^rd^ instars by *in situ* hybridization. Representative midgut sections (Supplementary Fig. 3) were from WCR 3^rd^ instars treated with H_2_O (top panel), *gus* dsRNA (middle panel) and *dvssj1* frag1 (bottom panel) at 50 ng μl^−1^ for 48-h. All treatments were hybridized with the *dvssj1* probe and an RNAscope^®^ negative control probe (*Bacillus subtilis* dihydrodipicolinate reductase (*dapB*) gene). Expression of *dvssj1* mRNA is observed in midgut epithelium cells (ep) and oenocyte cells (o) of H_2_O and control *gus* treatment. Knockdown of *dvssj1* mRNA in midgut epithelium cells (ep) and oenocyte cells (o) is observed in larvae treated with *dvssj1* dsRNA (bottom panel). No clear presence of *dvssj1* mRNA in fat body cells (f) and *dvssj1* dsRNA in midgut lumen was observed (arrow). Images were captured at 40× magnification with 100 μm scale bars.

Suppression of *dvssj1* mRNA accumulation was demonstrated using both quantitative RT-PCR and *in situ* hybrization (ISH) methods. Expression of *dvssj1* mRNA was quantified from insects exposed to 0.5 ng μl^−1^ of diet incorporated *dvssj1* dsRNA and collected after 12 and 48 h of feeding. *Dvssj1* mRNA accumulation was significantly (P < 0.05) reduced following *dvssj1* dsRNA treatment but not with a control dsRNA (*gus*) or water control (Fig. 3b). *Dvssj1* expression was lowest 48 h post treatment. Localization of *dvssj1* mRNA molecules in 3^rd^ instar WCR was demonstrated using RNAscope ISH. *Dvssj1* mRNA molecules were predominantly present in the cells of the midgut epithelium (Fig. 4) but were also detected in the oenocyte cells^33^. At 48 h post-treatment of *dvssj1* dsRNA, midgut epithelium cells showed a loss of *dvssj1* mRNAs. The distal oenocyte cells also showed a nearly complete loss of *dvssj1* mRNAs.

### Ultrastructure observations

**Figure 5:**
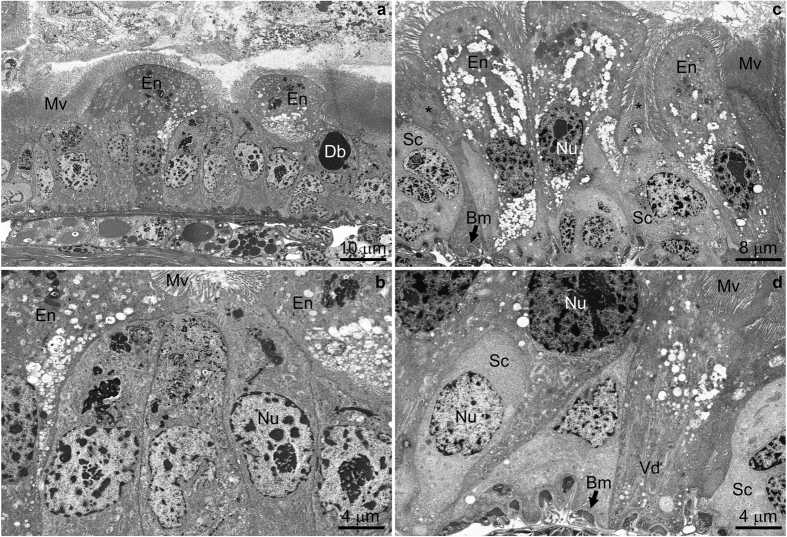
Electron micrographs of *dvssj1* dsRNA-treated neonates after 72 h exposure. (**a**) Anterior midgut epithelium exhibiting apoptotic enterocytes (En) that mostly lack basal extracellular labyrinth, but contain highly vesiculated cytoplasm. Numerous stem cells are present, typically containing electron dense cytoplasmic inclusions. A “dark body” (Db) consisting of nascent microvilli is taken as evidence of an active regenerative process. Mv, microvilli (**b**) Higher magnification of a central area taken from panel a to better illustrate stem cell detail. Nu, nucleus **(c**,**d)** Epithelium from anterior/middle and middle midgut regions. Note apoptotic enterocytes with highly vesiculated cytoplasm either lacking or with reduced, partially vesiculated (Vd) basal extracellular labyrinth. Regenerating cells (*) are interspersed among sloughing cells and stem cells (Sc). Bm, basal membrane; Non-osmicated specimens.

After 72 h of feeding, *dvssj1* dsRNA treatment (100 ng μl^−1^) resulted in an overall decrease in neonate length compared to untreated controls (Supplementary Fig. 4a,b); in some cases, the gut lumen volume was greatly reduced by the presence of apparent blebs from intestinal epithelial cells. Unique ultrastructural features very rarely or not observed in corresponding untreated controls (cf. Supplementary Fig. 5a–e) indicative of midgut epithelial cell injury were found in treated neonates at 72 h. These features included apparent manifestations of apoptosis and accelerated regenerative activities such as unusual stem cell morphology, reduction in basal extracellular labyrinth, and appearance of numerous vesicles in different regions of enterocyte cytoplasm (Fig. 5a–d).

### *Dvssj1* provides root protection against WCR

**Figure 6:**
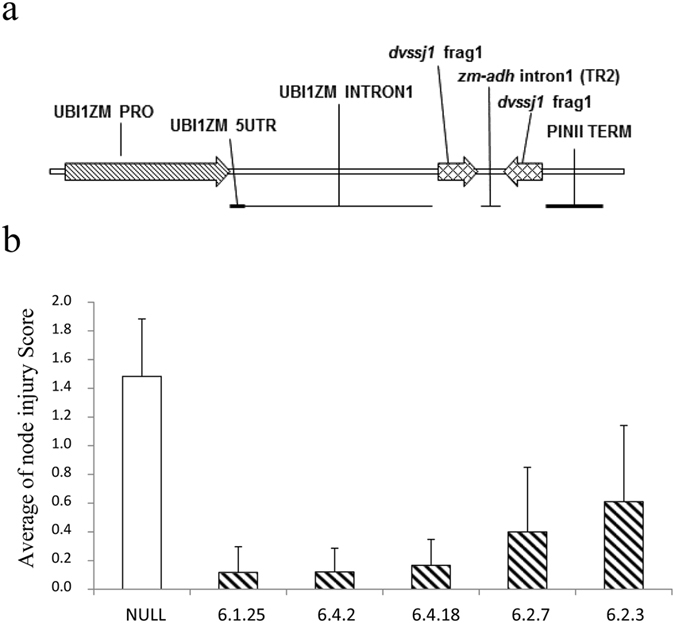
T1 plants expressing *dvssj1* dsRNA show root protection from WCR feeding damage. (**a**) Map of the *dvssj1* expression cassette. (**b**) Five *dvssj1* transgenic lines and one transgenic negative isoline (NULL) were selected for T1 greenhouse assay. Fifteen or fourteen plants per *dvssj1* dsRNA line and NULL plants were assayed for WCR feeding damage^34^. The node injury score (mean ± SD) was significantly different (p-value < 0.0001) between the NULL and all transgenic lines.

Transgenic maize lines expressing *dvssj1* dsRNA were generated through transformation of a Pioneer inbred line, PHR03. Five transgenic events expressing *dvssj1* dsRNA were selected for greenhouse assay at the T1 generation (14–15 plants per event) and infested with 1000 WCR eggs at the V6 (six-leaf) stage. Plants were scored for WCR feeding damage^34^ three weeks after infestation. The average node injury scores for the transgenic events were 0.12–0.61, which was a significant (P < 0.0001) reduction from the corresponding score of 1.5 for the negative control isoline (Fig. 6 and Supplementary Table 4).

**Figure 7:**
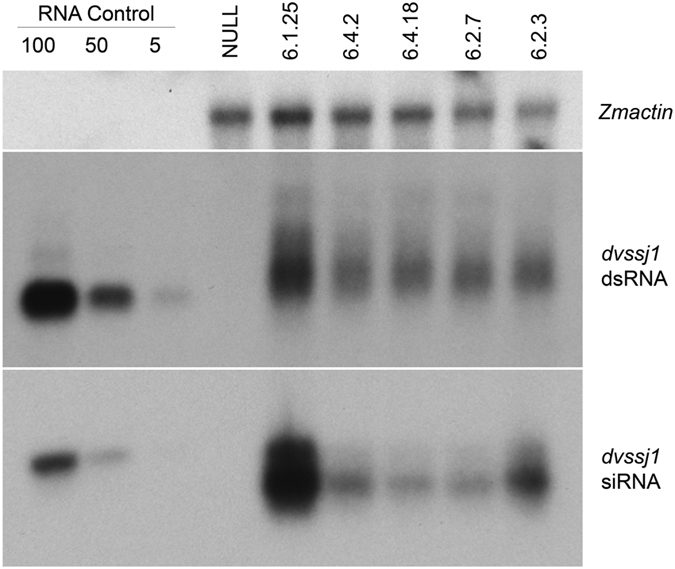
Northern blot analyses of T1 *dvssj1* root samples. Northern blots of five *dvssj1* containing events and one transgenic negative isoline (NULL). *Zmactin* (Accession #: EU952376; top panel) was included as a reference gene for northern analysis. Double-stranded *dvssj1* frag1 (210 bp; 100, 50 and 5 pg) RNAs were loaded as a positive control of dsRNA (middle panel); 29nt *dvssj1* oligo (100, 50 and 5 pg) was used as a positive control for the siRNA northern blot (bottom panel).

Total RNA was extracted from root tissues for northern blot analysis to examine RNA expression in the T1 transgenic plants. Two species of RNA were detected by the dsRNA northern blot (Fig. 7) - a dominant band migrating at approximately 232 nucleotides (nt) and a less intense band migrating higher than 232 nt. The 232 nt dominant band likely represents dsRNA (Supplementary Fig. 6b). DsRNA transcripts have previously been reported in maize^13^,^19^. *Dvssj1* dsRNA derived small RNAs (21 to 24-nt RNAs) were identified on a siRNA northern blot (Fig. 7). The prevalent species of siRNA appeared to be 21 nt fragments, consistent with previous findings in transgenic maize containing RNAi constructs^13^,^19^. Expression of *dvssj1* RNAs correlate to the copy number of the transgene and has an inverse relationship with nodal injury score (Fig. 7 and Supplementary Table 4).

## Discussion

In order to screen for new RNAi targets in WCR we developed a 7-day diet based assay. Two key factors were employed for an efficient and reliable diet assay: (1) DsRNAs were incorporated into standard WCR artificial diet containing food coloring to monitor feeding, and (2) only healthy larvae were selected for the screening. Using this assay, thirty-five RNAi active targets were identified (Supplementary Table 1a). These targets included members of several gene families that have been previously reported^13^,^17^, such as ribosomal proteins, proteasome subunits, transcription and translation initiation elongation factors^35^. Other notable RNAi gene targets included members of the small GTPase superfamily^36^, heat shock proteins, and actin genes^13^,^15^. Two unique genes, *dvssj1*^37^ and *dvssj2*^38^, initially identified as effective RNAi targets were subsequently found to be orthologs of *Drosophila ssk*^30^ and *mesh*^31^ proteins, respectively (Table 1, Figs 1 and 2). The first SSJ gene was reported in *Bombyx mori* (silkworm) using monoclonal antibodies that specifically recognized the apical region of the lateral membrane of midgut epithelial cells. The monoclonal antibodies were subsequently used to immunoprecipitate proteins from a midgut membrane fraction followed by protein sequence determination using mass spectrometry^30^. The *Drosophila* ortholog was identified by homology search and named Snakeskin (*ssk*). SSK has four membrane-spanning domains predicted in its primary protein sequence. Another SSJ gene, *mesh*, was also identified using a similar approach^31^. Proteins of both genes are important to the formation of the SSJ in the *Drosophila* midgut^28^.

In *Drosophila*, SSK and MESH colocalize to SSJ and are specifically expressed in endodermally derived epithelia, including the midgut and gastric caeca^30^,^31^. Western analyses confirmed that both DVSSJ1 and DVSSJ2 were present in WCR midgut derived tissues (Fig. 3a). Several higher MW bands were visible in the DVSSJ1 western blot which were likely non-specific signals, protein complexes with DVSSJ2^31^ or complexes with other unidentified proteins. SSK expression appears at stage 12 of *Drosophila* embryos in midgut rudiments as a protein band of ~15 kDa and its expression is sustained until the adult stage throughout the midgut and Malpighian tubules^30^. The lower MW band in the DVSSJ2 western blot may represent truncated version or a form of split variant. There are five different *mesh* variants in Flybase^39^, which translated into three isoforms with different C-terminal cytoplasmic regions. MESH was detected by western analysis as a main ~90 kDa band and a minor ~200 kDa band^31^. Compromised *mesh* expression causes defects in the organization of SSJs, resulting in the mis-localization of other SSJ proteins, and the loss of barrier function of the midgut. Ectopic expression of MESH in cultured cells induces cell-cell adhesion. *Drosophila* SSK and MESH form a complex together and these proteins are mutually interdependent for their correct localization in SSJ formation^31^.

Quantitative RT-PCR analyses confirmed that ingestion of *dvssj1* dsRNA resulted in suppression of *dvssj1* mRNA (Fig. 3b). *Dvssj1* mRNA expression patterns and *dvssj1* mRNA knockdown were also demonstrated in 3^rd^ instar WCR using RNAscope ISH (Fig. 4). *Dvssj1* only expressed in midgut epithelium cells and oenocyte cells, and expression patterns varied slightly between different regions of the midgut (Supplementary Fig. 3). The gene expression of *dvssj1* mRNA in midgut epithelium cells of WCR corroborates the functional role of *dvssj1,* analogous to *Drosophila ssk*^30^. *Dvssj1* mRNA were also detected in oenocyte cells, which are cells responsible for lipid processing and detoxification^40^.

The physical integrity of the SSJ is important for controlling the paracellular pathway between epithelial cells, which effectively separates the gut lumen, where digestion occurs, from the interstitial space, where metabolites and electrolytes are tightly regulated^25^. The SSJ is composed of a group of proteins physically connecting adjacent cells and contribute to the specialization between epithelial cell apical and basolateral membranes^28^. Although the molecular architecture of the WCR SSJ has not been fully characterized, DVSS1 protein is clearly an ortholog of the integral membrane protein (SSK) in *Drosophila*^30^. Mutant flies lacking *ssk* do not survive early larval development^28^. Flies with reduced *ssk* expression exhibit deformed midgut epithelial cells and uncontrolled leakage of a tracer dye from the gut into the hemocoel^30^. Similarly, suppression of *mesh* has the same effect on fly midgut epithelium^31^. The extracellular domains of MESH are found in cell adhesion proteins that are involved in cell-cell and cell-matrix adhesion^28^,^31^. The toxic effect to WCR resulting from oral exposure to *dvssj1* dsRNA in diet or expressed *in planta* can be attributed to suppression of *dvssj1* mRNA leading to reduction in DVSSJ1 expression/accumulation, loss of the midgut epithelium diffusional barrier, and cellular deformities due to improper intercellular contacts. Future studies may include quantitative analyses of *dvssj1* mRNA and protein from insects exposed to different doses of dsRNA to help understand *dvssj1* RNAi effects and target protein stability or turnover rate.

Cytological observations of nearly whole neonates in section (Supplmentary Fig. 4) showed a significant difference in the overall size between treated and untreated individuals, as well as an apparent occlusion of the gut lumen, and numerous examples of enterocyte blebbing into the gut lumen, after *dvssj1* dsRNA consumption. Extensive comparison of dsRNA-treated neonate sections to sections prepared from untreated controls was important for distinguishing between normal cell regeneration and molting, and potential effects of *dvssj1* dsRNA treatment on neonate mid-gut epitheial cell ultrastructure. For example, dark bodies that contained what appeared to be nascent microvilli^41^ were very often observed at 72 h in dsRNA-treated neonate samples (Fig. 5), but rarely in the controls (Supplementary Fig. 5). In gut areas, where much blebbing of enterocyte cytoplasm into the gut lumen could be observed, basal extracellular labyrinth (Bl) was sometimes not evident. Such regions also exhibited enlarged and differentiating stem cells which we interpreted as evidence of active molting or possible stress response^42^. These regions, which also bore additional subcellular markers such as dark bodies as mentioned above, and highly vesiculated cytoplasm, were especially prevalent in *dvssj1* dsRNA-treated larval gut, and only rarely observed in controls. These observations are consistent with the notion that suppression of *dvssj1* expression and its protein accumulation are the cause of WCR growth inhibition and mortality.

SSK and MESH are improtant to the formation of the SSJ in the midgut of *Drosophila*^30^,^31^. *Ssk* orthologs have been identified in other arthropods, but not in vertebrates^30^, suggesting that SSJs composed of MESH and SSK are arthropod-specific cell–cell junctions. However, MESH homologs are present in other metazoans, including *C. elegans*, sea urchins and mammals^31^. A sequence search of public and internal databases suggests that *dvssj1* orthologs are only found in arthropods and not in vertebrate species or maize (Supplementary Fig. 6 and Supplementary Table 5). This makes *dvssj1* a good target for applying RNAi rootworm control in transgenic plants.

*Dvssj1* dsRNA targets the expression of a protein important for the formation of SSJ between epithelial cells lining the midgut. Ingested *Dvssj1* dsRNA has a relatively fast biological effect on WCR as indicated by its short LT_50_ (Table 2) which may be a consequence of direct exposure of midgut epithelial cells to dsRNA. Consequently, the biological effect of *dvssj1* RNAi may have no dependency on systemic movement of the silencing signal^11^. During WCR larval development, the midgut epithelial surface area grows by the continuous increase of the number of cells^43^. Maintenance of midgut epithelial characteristics during this period requires tightly regulated SSJ to support vital structure and barrier functions. Disruption of SSJ by the down regulation of *dvssj1,* makes this a well-suited gene target for RNAi silencing and an alternative “mode of RNAi action” for the control of corn rootworm. Under greenhouse conditions, mean node injury scores for the transgenic *dvssj1* events ranged from 0.12–0.61, which was a significant (P < 0.0001) reduction from the corresponding score of 1.50 for the negative control isoline (Fig. 6 and Supplementary Table 4). Dun *et al*.^44^ and Tinsley *et al*.^45^ estimated that under field conditions, one node of root injury was on average associated with a corn yield loss of approximately 15–18%. Further studies are needed to confirm the efficacy^46^ of *dvssj1* events under field conditions^47^.

## Conclusion

The discovery of *dvssj1* and *dvssj2* genes in WCR provides new potential gene targets or “modes of action” at the gene level for the control of this important pest using RNA interference technology. Double-stranded RNA targeting *dvssj1* expressed in transgenic maize plants can effectively down regulate the expression of the *dvssj1* gene in WCR larvae, leading to larval growth inhibition and mortality.

## Methods

### WCR cDNA library and identification of RNAi active clones

The cDNA library construction kit from Clontech (Mountain View, CA) was used to make WCR cDNA libraries. Total RNA was extracted from WCR neonates or 2^nd^–3^rd^ instars and cDNAs were cloned into the Sfi I site of the pDNR-LIB library vector according to the manufacturers’ instructions. Expressed sequence tag (EST) sequencing was performed using Applied Biosystems capillary sequencers. RNAi target screening includes primary screening (8 replicates for each target) and confirmation (8 replicates per target) round, which was conducted on subset of primary active targets based on primary score (activity) and target novelty. Dose response assays were used for further characterizing insecticidal activities in diet. After an active target (cDNA clone) was identified *via* tBLASTx against both *Tribolium* and/or *Drosophila* database, full length cDNA was sequenced using standard Sanger sequencing methods or transcript was identified from the WCR transcriptome analysis (Supplementary Table 1a). Sequence alignment was derived using CLUSTAL W with default parameters^48^. Protein domains were predicted by the Pfam protein families database^49^. The transmembrane domains with hydrophobic residues were predicted by the SOSUI algorithm^50^.

### Double stranded RNA production by *in vitro* transcription

To screen WCR active targets, 400 to 800 base pair regions of randomly selected non- redundant cDNA clones were amplified using Taq DNA polymerase with a pair of gene specific primers (Supplementary Tables 1b and 2). The gene-specific primers also contained T7 RNA polymerase sites (5′d[TAATACGACTCACTATAGGG]3′) at the 5′ end of each primer. PCR product served as the template for dsRNA synthesis by *in vitro* transcription (IVT) using a MEGAscript kit (Life Technologies, Carlsbad, CA). DsRNAs were examined by 48 well E-gel electrophoresis (Life Technologies) to ensure dsRNA integrity and quantified using Phoretix 1D (Cleave Scientific).

### WCR bioassays

#### Diet-based bioassays for primary screening

WCR diet was prepared according to the manufacturer’s guideline for *Diabrotica* diet (Frontier, Newark, DE) with modifications^51^. DsRNA samples were incorporated into diet at 50 ng μl^−1^ final concentration in a 96 well microtiter plate format. In each well of the plate, a mixture of 5 μl of dsRNA (300 ng μl^−1^) and 25 μl of WCR diet were added to each well of the plate and shaken on an orbital shaker for 1 minute until the diet solidified. Eight replicates (wells) were used for each RNA sample. Preconditioned 1^st^ instar WCR (neonates were placed on diet for 24 h prior to transfer to the test plate) were added to the 96 well plates; 2 insects per well. After 7 days of incubation, larvae were scored for growth inhibition and mortality using the following scale: 0 = No effect, larvae are equal to control plate larval growth (2^nd^ instars), 1 = Slight larval stunting, larvae are slightly smaller (i.e ~25% reduction) in length and width, 2 = Severe larval stunting, larvae are 1st instars (approx. the size of the infested neonates or >60% reduction in size of healthy insects), 3 = Dead (100% Mortality). The primary and confirmation scores were based on an average score across all eight replicates.

#### LC_50_ and IC_50_ determination

Double-stranded RNAs were incorporated in diet as described for primary screening. For each sample, ten doses (100, 31.6, 10, 3.16, 1, 0.316, 0.10, 0.032, 0.010 and 0.0032 ng μl^−1^) were evaluated for a total of 32 observations per dose or water control. Four plates were employed with 8 wells on each plate for each concentration. Two one-day old larvae were transferred into each well. Plates were incubated at 27 °C and 65% RH. Seven days after exposure larvae were scored for growth inhibition (severely stunted larvae with >60% reduction in size) and mortality. Data were analyzed using PROC Probit analysis^52^ in SAS to determine the 50% lethal concentration (LC_50_). The total numbers of dead and severely stunted larvae were used for analysis of the 50% inhibition concentration (IC_50_).

#### Lethal Time (LT_50_) determinations

LT_50_ assays were performed on five WCR dsRNA target genes, with two different assays for each sample. In the first assay, the same method as was described for the LC_50_/IC_50_ determinations was used. A single one-day old larva was transferred into each well of a 48 well plate containing 400 μl of diet with dsRNA sample at 5 ng μl^−1^. In the second assay, a single one-day old larva pretreated on diet containing 50 ng μl^−1^ of dsRNA was transferred into each well of a 48 well plate containing 400 μl of diet and dsRNA at the same dose for each sample. The plates were scored daily for mortality 1–12 days after infestation. The Weibull distribution for Survival analysis in SAS (Version 9.4) was used to describe the time to mortality curve. Each insect was treated as an individual data point for the LT_50_ output based on a Weibull distribution. LT_50’_s were considered significantly different if 95% confidence intervals (CI) (*P* < 0.05) were non-overlapping.

#### Plant expression vectors and transformation

Standard DNA and RNA techniques as described by Sambrook and Russell^53^ were used for vector construction and expression analyses. To demonstrate rootworm efficacy *in planta*, a fragment of the *dvssj1* gene was assembled into a suppression cassette designed to express dsRNA targeting a section of the *dvssj1* gene. The silencing cassette consisted of the maize ubiquitin promoter, maize ubiquitin intron 1^54^, two 210 base pair stretches of *dvssj1* and an intervening truncated maize ADH intron1 designed to support assembly into a dsRNA hairpin (Fig. 6a and Supplementary Fig. 6b), and the PIN II terminator. The *dvssj1* construct was transformed via *Agrobacterium tumefaciens* into a commercial maize elite-inbred line, PHR03^55^. T0 maize transformants were screened by qPCR analyses^56^ and transferred to soil and backcrossed with a PHR03 inbred line to generate T1 progeny.

#### Greenhouse WCR feeding assays

T1 plants containing one or more copies of the *dvssj1* silencing cassette were evaluated using a greenhouse assay to assess rootworm feeding damage. T1 seeds were planted in 32-cell flats containing Fafard Superfine potting mix. Fifteen or fourteen PCR positive plants (Supplementary Table 4) at growth stage V2–V3 were transplanted into pots containing approximately 4.5 liters of SB-300 potting mix. At 25 days post-planting, the root zones of plants were infested with 1000 WCR eggs per pot. Twenty one days after infestation, individual plants were scored using the 0 to 3 root node-injury scale developed by Oleson *et al*.^34^. Negative controls consisted of a transgenic PHR03 null isoline. Statistical calculations were performed using JMP (Version *12*. SAS Institute Inc., Cary, NC). To assess the root protection from corn rootworm feeding provided by *dvssj1* in transgenic plants, the data were analyzed by non-parametric one-way ANOVA (Kruskall-Wallis; P < 0.0001). Dunnett’s *post hoc* test was used to compare all treatments against the control treatment, NULL.

### DsRNA and siRNA northern blot analyses

Total RNA was extracted using the mirVana™ miRNA Isolation kit (Life Technologies, Carlsbad, CA) from T1 transgenic maize plants at leaf stage V6–V7. Ten μg of total RNA was fractionated on a 1.5% denaturing formaldehyde gel. For siRNA northern blot analysis, 20 μg of total RNA was fractionated on a 15% Criterion™ TBE-Urea Gel (Bio-Rad, Hercules, CA). RNAs were blotted to a Hybond-N+ membrane (Amersham, Little Chalfont, United Kingdom). The blots were pre-hybridized in ExpressHyb™ hybridization solution (Clontech, Mountain View, CA) for 1 h and then hybridized in the same solution as the DNA probe overnight at 65 °C for dsRNA and 37 °C for siRNA, respectively. The autoradiographs were digitized by ImageQuant™ LSA4000 (Fujifilm, Tokyo, Japan). Densitometry analyses of northern blots was peformed using Phoretixs 1D software (Cleaver Scientific, Rugby, UK).

### Quantitative real-time PCR (qRT-PCR) and *in situ* hybridization (ISH)

Total RNA was extracted using Trizol and DNase I was used to remove genomic DNA. cDNA was synthesized from total RNA using the Bioline Sensifast cDNA kit (Taunton, MA) according to the manufacturer’s instructions. The designs of primers and probe regions are listed in Supplementary Table 3. *Dvssj1* gene expression was quantified from WCR larvae collected after 12 and 48 h of feeding on diet incorporated with 0.5 ng μl^−1^ of *dvssj1* frag1 dsRNA. Gene expression was analyzed using two-step real time quantitative RT-PCR. The assay was run, with 3 replicates per sample, using a single plex set up with Bioline Sensifast Probe Lo Rox kit (Taunton, MA) and analyzed using the 2^−ΔΔCt^ method based on relative expression of the *dvssj1* gene and a reference gene *dvrps10*. Data from qRT-PCR assays were analyzed using JMP (Version *12*. SAS Institute Inc., Cary, NC) and statistical differences were detected using one-way analysis of variance (ANOVA) followed by Dunnett’s post-test; *P* < 0.05 was considered statistically significant.

For ISH analyses, target probes, preamplifier, amplifier, and label probe were designed by Advanced Cell Diagnostics (Hayward, CA). For chromogenic detection using 3,3′-Diaminobenzidine (DAB), label probe was conjugated to horseradish peroxidase (HRP). WCR were reared on artificial diet until 3^rd^ instar,and then moved into individual wells containing diet incorporated with a *dvssj1* frag1dsRNA and control dsRNA *gus* at 50 ng μl^−1^. Insects were collected 48 h post-treatment, fixed in 10% neutral buffered formalin (4% formaldehyde) for 48 to 72 h and processed for paraffin embedding. Paraffin sections were cut 4 μm thick, collected on Superfrost Plus slides (Fisher Scientific), air-dried overnight, and baked for 1 h at 60 °C. Sections were processed for RNA *in situ* hybridization with the RNAScope Detection Kit (Chromogenic) according to the manufacturer’s standard protocol (Advanced Cell Diagnostics, Hayward, CA). Slide images were acquired using a Leica Aperio^®^ AT2 digital scanner and captured at 40× magnification with resolution of 0.25 μm pixel^−1^.

### Western blot analysis

Western blot analysis was performed on solubilized WCR gut extracts from 3^rd^ instar. The dissected gut tissue proteins were homogenized in buffer (50 mM Na2HPO4-NaH2PO4, 50 mM NaCl, 5 mM EGTA, 5 mM EDTA, pH 7.5) containing 2% Triton X100, 2 complete protease inhibitor cocktail tablets EDTA-free (Roche, Basel, Switzerland), and 1mM PMSF. The proteins were electrophoretically transferred to a nitrocellulose membrane and detected with either anti-DVSSJ1 mouse Ab (1:2,500, Genscript, USA) or anti-DVSSJ2 hybridoma supernatant mouse Ab (1:50, Genscript, USA), followed by goat anti-mouse (GAM)-HRP conjugated secondary Ab (Bio-Rad, Hercules, CA) (1: 12,500).

### Microscopy

WCR eggs were reared on the same diet used for primary screening. Double-stranded RNA of *dvssj1* frag1 was labelled with Cy3 by IVT and Cy3 fluorescence was used to confirm WCR feeding at a final concentration of 100 ng μl^−1^. Untreated control neonates were prepared by adding an equivalent volume of dsRNA buffer solution containing Cy3 only (no RNA). WCR neonates were transferred singly into wells at 24 h post-hatch and were collected after 72 h incubation with dsRNA and prepared for electron microscopy as described in the Supplementary Methods and by Rizzo *et al*.^57^ who have reported techniques for examination of ultrastructure via backscattered electron imaging in a scanning electron microscope.

## Additional Information

**Accession codes**: The RNAi active target sequences have been deposited in the GenBank of National Center for Biotechnology Information under the accession number KU562965 (*dvssj1*); KU562966 (*dvssj2*); KU756279 (*dvprotb*); KU756280 (*dvpat3*) and KU756281 (*dvrps10*).

**How to cite this article**: Hu, X. *et al*. Discovery of midgut genes for the RNA interference control of corn rootworm. *Sci. Rep.* **6**, 30542; doi: 10.1038/srep30542 (2016).

## Supplementary Material

Supplementary Information

## Figures and Tables

**Table 1:** Diet-based results of WCR dsRNA screening.

dsRNA Name	Length (bp)	Relative to orf		Primary scores	7d LC_50_, ng μl^−1^	7d IC_50_, ng μl^−1^
		Start	End			
*dvssj1* FIS	1156	−27	1131	2.8	n/a	n/a
*dvssj1* frag1	210	−25	185	2.9	0.041	0.013
*dvssj1* frag2	145	−6	139	3.0	0.097	0.013
*dvssj1* frag5	502	−25	477	2.0	0.082	0.022
*dvssj2* FIS	573	2934	3506	3.0	1.699	0.272
*dvssj2* frag1	225	3301	3526	2.6	0.286	0.135
*dvssj2* frag7	162	16	177	2.4	0.089	0.054

Primary scores were the average of eight observations in cDNA-based first-round IVT screening (FIS) or subsequent fragment screening. LC_50_ and IC_50_ values in ng μl^−1^ during a 7-day assay. Target sequences are indicated relative to the first letter of the start codon (**A**TG) of the open-reading frame (orf).

**Table 2:** LT_50_ results of five dsRNA’s in WCR.

Dose (ng μl^−1^)	dsRNA	% Mortality (12 days)	LT_50_ (days)	95% CI	Grouping*
50	*dvssj1* frag1	100	5.5	5.2–5.8	a
	*dvssj2* frag7	100	6.3	6.0–6.7	b
	*dvprotb* frag1	97.8	8.5	7.9–9.0	c
	*dvpat3* frag13	93.5	9.0	8.4–9.7	c
	*dvrps10* frag4	97.8	9.0	8.5–9.7	c
5	*dvssj1* frag1	100	6.6	6.2–7.1	a
	*dvssj2* frag7	100	7.1	6.7–7.6	a
	*dvprotb* frag1	97.9	8.4	7.8–9.0	b
	*dvpat3* frag13	91.7	9.2	8.6–10.0	bc
	*dvrps10* frag4	83.3	10.0	9.3–10.7	c

LT_50_ represents time in days that 50 percent of WCR larvae (n=45–48) are killed by dsRNA at concentrations of 5 or 50 ng μl^−1^. Fragments of five active targets were selected for LT_50_ determination based on dose response assay results (Table 1 and Supplementary Table 2). LT_50_ values with different letters are significantly different based on non-overlap of 95% CI (P < 0.05).
