# Supplementary material for: Discovery of midgut genes for the RNA interference control of corn rootworm
Source: Sci Rep. 2016 Jul 28;6:30542. doi: 10.1038/srep30542 (PMC4964579; doi:10.1038/srep30542)
Supplement: Supplementary Information [file srep30542-s1.pdf]

Supplementary information

Discovery of midgut genes for the RNA interference control of corn rootworm

Xu Hu[1*], Nina M. Richtman[1], Jian-Zhou Zhao[1], Keith E. Duncan[2], Xiping Niu[1], Lisa A. Procyk[1], Meghan A. Oneal[1], Bliss M. Kernodle[1], Joseph P. Steimel[1], Virginia C. Crane[1], Gary Sandahl[1], Julie L. Ritland[1], Richard J. Howard[2], James K. Presnail[1,3] Albert L. Lu[1], and Gusui Wu[1]

[1]DuPont Pioneer, Johnston, IA, USA. [2]DuPont Pioneer, Wilmington, DE, USA.

[3]Present address: Evogene Ltd., Saint Louis, MO, USA.

[*]Corresponding Author: Xu Hu, DuPont Pioneer, 7300 NW 62nd Ave. Johnston, IA 50131, USA

Telephone: 1-(515)-535-2105

E-mail:xu.hu@pioneer.com

Supplementary Figure 1. Protein alignments of DVSSJ2 and *Drosophila* MESH.

```
DVSSJ2        MWFKYFVIFVLFS-----------VSVKGLDGDLPPDV-ETIDVETTNVSNADPDAGKPI
MESH          MRFKLFVVCALACGFMAFVLANENISTEEFEDQIINAVPEPVKVTKPKAQAEFVEVPKAT
              * ** **: .* .           :*.: ::.::   * *.:.* ..:..    :. *.

DVSSJ2        PVEDTPNGVPPVNTNYEVDKS----LVNVTNS-----LVNVTKNPRRTYQTVNKMGKYS-
MESH          PAPKKAEADNPKTKPLALQKPTLIPVVHVSSRGTDDILTVAGLKPEKMFGALIMPNDYLG
              *. ...:.  * ..   ::*.    :*:*:.      *. .  :*.: : ::   ..*

DVSSJ2        TFYAADYDPMTSDT----APPDTDQRGGGGTPYTISEPRLAEIRKHFMYPFFDKGGSDNN
MESH          ELYDVDNSGYNWDPNNNVAPPTTYSTAAGG--YTITAARLAELRSNFMYWYFDKDMYGG-
               :* .* .  . *.    *** * . ..**  ***: .****:*.:*** :***.  ..

DVSSJ2        LGDFQKDIQSSIPQVHKNLNFQLPFFGFRYNYTRVSVNGYLEFSDPPPNYDYPLVFPIKE
MESH          RGDYQFDIHASMTQLHKNLNFQLPFYGFRFNYTRLSLNGYLEFSDPPEYLTYPLVFPIKD
               **:* **::*:.*:**********:***:****:*:**********    ********:

DVSSJ2        WPKKNDPAFIGIFFSRCRIGNLRDGDIDQRTPGVYFRMERDLRTRQDRMGVEIRERLKWD
MESH          WPAKRDPSFMGIFFSKCRVGRIYPSDIDQRTPGVYFRVERDLMGRTDRFGVEVRERTMWD
              ** *.**:*:*****:**:*.:  .************:****  * **:***:***  **

DVSSJ2        IRTGVIGSETFDPKHAVIVTWKNVTFTGGFANAKYKTNTFQMVLATDEVFTYAMFNYLNL
MESH          IRQGVVGADTFIPKHVVIATWKNVSFAGGIDNSLYTTNTFQMVLATDEVYTYIIFNYAVL
              ** **:*::** ***.**.*****:*:**: *: *.*************:** :***  *

DVSSJ2        DWTSHTEAGGDSQNGEGGVSAYVGFNAGNGTRSYEYNPYSQASVIRDLTSVGFGNGFKGR
MESH          NWLSHTEAGGDTTKGEGGVPAYVGFNAGNGTQAYEYNPYSQNMVIRDLANRGWANGFPGR
              :* ********: :*****.***********::********  *****:. *:.*** **

DVSSJ2        HIFRIDEDILLGSCNKDIDGANLPLKFAPESGNMLGGTVVNITGPCFNLNDRIRCKFDVS
MESH          HIFRVDEQILIGSCNKDIDAALLPLTFAPESGNMLGGQVVNITGPCFDPAIRVTCHFDT-
              ****:**:**:********.* ***.*********** *********:   *: *:**.

DVSSJ2        NEVFGYVVDKNRAICVQPQLYAEGWVNLQIAVNSEAFKWKGKYYVESPASATQKIFFKDM
MESH          ESVLGTYVDRNRVICVQPYLKAEGYIRFQISVGTQRFKWRGKYFVETPAAATEKIFFTTD
              :.*:*  **:**.***** * ***::.:**:*.:: ***:***:**:**:**:****.

DVSSJ2        KLHEKSPSELKITWEKQNLTTNDNANIRISLWGYRETTMKPVFVYITDIADNVQNIGEHT
MESH          DVHKKNPAEIRITWNQYNLTSNANANVMISLWGYRETKIEPQLEYIDVIEASYSNSGSYV
              .:*:*.*:*::***:: ***:* ***: *********.::* : **  *   . .* *.:.

DVSSJ2        IVPSQFRTRVNQYLTDIKFGFLQINLTESITVNTYTTSQSSIELTPVIWSRPIPLGWYFQ
MESH          ITPSNYINRNNINR-DMQFGFLQINLTQ-------PDQYSGLAISPVLWSRPIPLAWYMA
              *.**:: .* *    *::*********:       . . *.: ::**:*******.**:

DVSSJ2        FQWENQYGKNWPKYLCDDWLRTDRYLKNFAHELAQCPCTVEQALADKGRFMPDFDCDKDS
MESH          PQWERQHGKRWARALCDNWIRADRFLRNFAADLPLCPCTLDQAVLDKGRFRPDRECDKDS
               ***.*:**.*.: ***:*:*:**:*:*** :*. ****::**: ***** ** :*****

DVSSJ2        NPICYYNNQALHCVKTGSPTLEGSEQQCCYDKNGFLMLSYDQQWGSSPRRCHNLGKMPYN
MESH          NPSCLRHRGAIHCVVSGTPVAQGAEQQCCYDRYGFLMLTYDQMWGSRPRRVHNLGKMPWN
              ** *  :. *:*** :*:*. :*:*******: *****:*** *** *** *******:*

DVSSJ2        EATKVPTLSQWFNDMVPKYLCCLWQDEQAVGCETLRFERRPTQDCVAYQAPGVAGVYGDP
MESH          EASKVPSLSMWFHDMRPFYSCCYWQEEQAVGCETYRFERRPSQDCVAYQAPGIAGVFGDP
              **:***:** **:** * * ** **:******** ******:**********:***:***

DVSSJ2        HFVTFDDVEYTFNGKGEFALVKSVTQTDNLEIQGRFEQMDPNTYGEVRATQLTSVVAKGN
MESH          HFVTFDGTAYTFNGLGEFVLARSVDESNKFEVQGRFEQLPVNYYGEVKATQLTAVAMRGN
```

```
             ******.. ***** ***.*.:** :::::*:******:  * ****:*****:*. :**

DVSSJ2       NTIAIEVRRRPLDSRWRYRLDVIADNRRLYFDRPSLKFQHFQGVTIYTPSYILNQSEVII
MESH         TTTTIEVRLRPLHARWRYRLDVLADGRRVYFDRESLKFQHFDGVTVYTPTYLLNQSQVVV
             .* :**** ***.:********:**.**:**** *******:***:***:*:****:*::

DVSSJ2       MFDNGAGMEVVDNQGFMSARVFLPWSFINKTIGLFGNWSFNKEDDFTLPDGSRAAIVNNI
MESH         QFDAGIGVEVVENEGYMTGRVFLPWKFINKTAGLFGNWSFNKLDDFMLPNGQVAQLN--L
              ** * *:***:*:*:*:.******.***** ********** *** **:*. * :   :

DVSSJ2       NDMERVYNDFGSKWMLEDVLDPQKGRALFHREFGRTSATYNNKTFKPQFLMNPEDFLPTN
MESH         NDLRSIHTNFGIKWMLTDREVPGVGAALFKREFGRMSGYYANATFQPNYVLDPADFLPAN
             **:. ::.:** **** *   *  * ***:***** *. * * **:*:::::* ****:*

DVSSJ2       RSTDLKRISEICPLKLYECYYDYAMTVDRDLAHYTKNYKATIYQYKETTRRKVVSCGVLE
MESH         RSYDLERAEELCG-ECMQCQYDYAMTLNRDLAHFTKNYYDTIVNMQAENAVRVVSCGVLQ
             ** **:* .*:*  :  :* ******::*****:****  ** : :  .  :*******:

DVSSJ2       TPRFGRKSTFLFVPGTKVTYECIQEFVLVGDQRRECQADGTWNIPEYGYTYCLRQEEYSS
MESH         TPRFGRKLSFDFMPGAKVSFECNEGFILIGDQRRECLSNGLWNIPEYGYTECLREVYYTR
             ******* :* *:**:**::** : *:*:******* ::* ********* ***:  *:

DVSSJ2       RQAAITSGIILAIIIPLVLLLGYVGYKIYERLTSNNQNYDYDTVQKTQN-LQQFSRTLSP
MESH         RVAFIAIGIIFLVICPLMLCIVCGVYRYRQKQLKEDPQWQMPMLPRSRASSARNLRTLNY
             * * *: ***: :* **:* :    *:  ::  .:: :::   : :::    :  ***.

DVSSJ2       YREEDEDE---------KYPQSDTDSLSKK--------RRSYDKSYRTHEPLPNRPNS--
MESH         DDDSDTDASTLKKTKKWDLDENDEDVTSSEGEKPKQLGRRSLHSASGTELDQHGSPVDGD
               :.* *          .  :.* *  *.:        *** ..:  *.    . * .

DVSSJ2       ------EFEEKPLDP-------YDQTFEDD------RVSRTPTGS----PTSPTSSIQYT
MESH         HPDEDDDFDEADVHTGTTHPVGYHQPLSPEEADQLHRQQYSPTFSGLDSRTSGASSINYT
                   :*:*  :..       *.*.:. :      * . :** *     ** :***:**

DVSSJ2       TPYSRPDIIRGN---SSKNLNYN----DMYAEPIK-----------KPKERISASQSSIV
MESH         PQAATQNRFGGVPVLPSNTQYYSRPPSSVNTVPLRTAPTPLTQDTGLPSPPLATSPTPSV
             .  :  : : *    .*:.  *.    .: : *::            *.  :::* :. *

DVSSJ2       TDV---
MESH         QKSTEV
```

The predicted transmembrane domains with hydrophobic residues are indicated by underline. DVSSJ2 (MTSDTAPPDTDQRG 108-121 a.a.; bold) peptide was used for monoclonal antibody production. This alignment was derived using CLUSTAL W with default parameters[51]. * (asterisk) represents identical amino acid residues shared between DVSSJ2 and MESH, : (colon) conservation between two amino acid residues of strongly similar properties and . (period) indicates conservation between two amino acid residues of weakly similar properties.

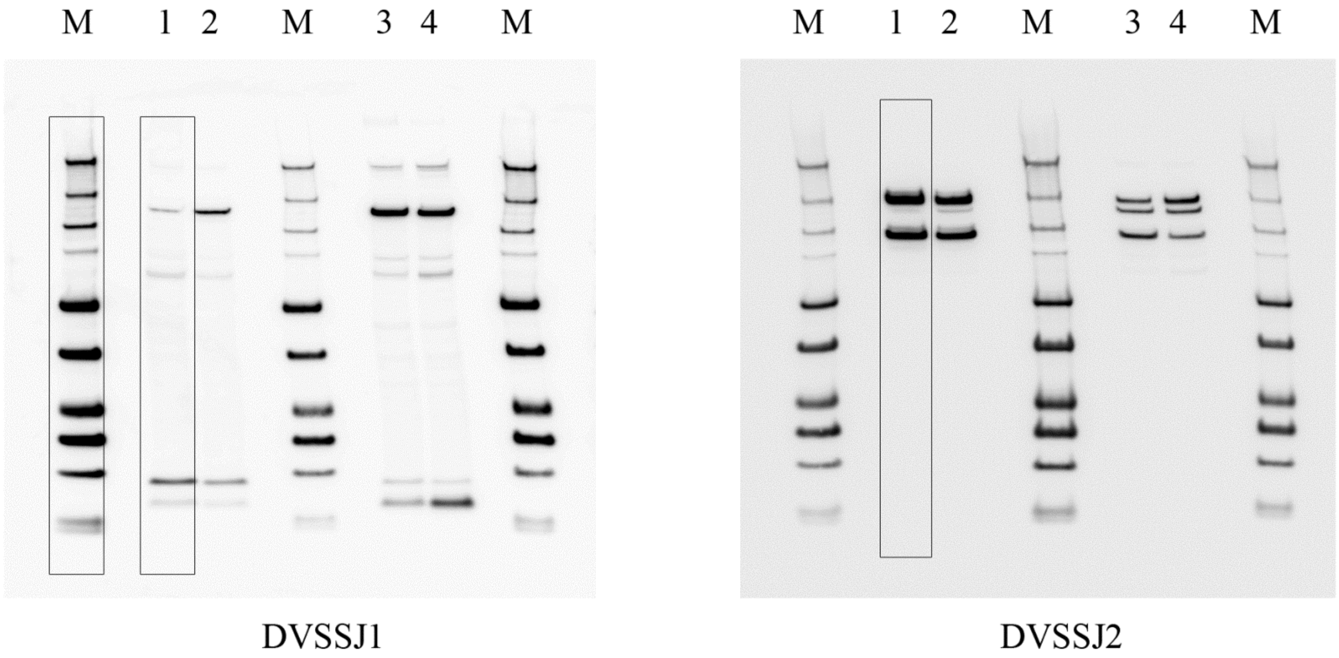

Supplementary Figure 2. Original pictures of western blots presented in results (boxes in Figure 3) to detect DVSSJ1 and DVSSJ2 from 3rd instar WCR dissected gut tissues.

Loaded protein samples represent the equivalent of one midgut (1) and 1/2 midgut (2). Protein samples (3 & 4) were extracted from midgut in 0.01 M phosphate buffered saline (NaCl 0.138 M; KCl - 0.0027 M); TWEEN® 20 - 0.05%, pH 7.4, at 25°C. The DVSSJ1 and DVSSJ2 detectable protein sizes are compared by Precision Plus Protein Western Standard (M) ranging from 10-250 kDa.

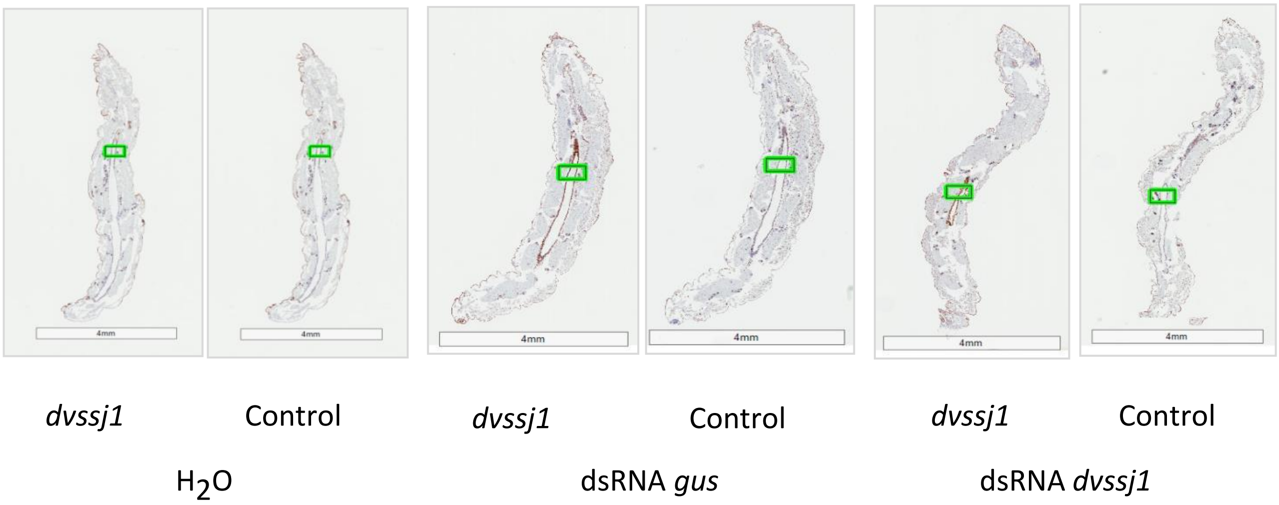

Supplementary Figure 3. Representative whole sections selected from ISH study.

WCR 3rd instar treated with H_2O, dsRNA *gus* and *dvssj1* frag1 at 50 ng µl^{-1} for 48-hr. All treatments (two adjacent sessions) were hybridized with *dvssj1* probe and RNAscope® negative control probe (*Bacillus subtilis* dihydrodipicolinate reductase (*dapB*) gene). Only selected regions (box) were presented in results (Figure 4).

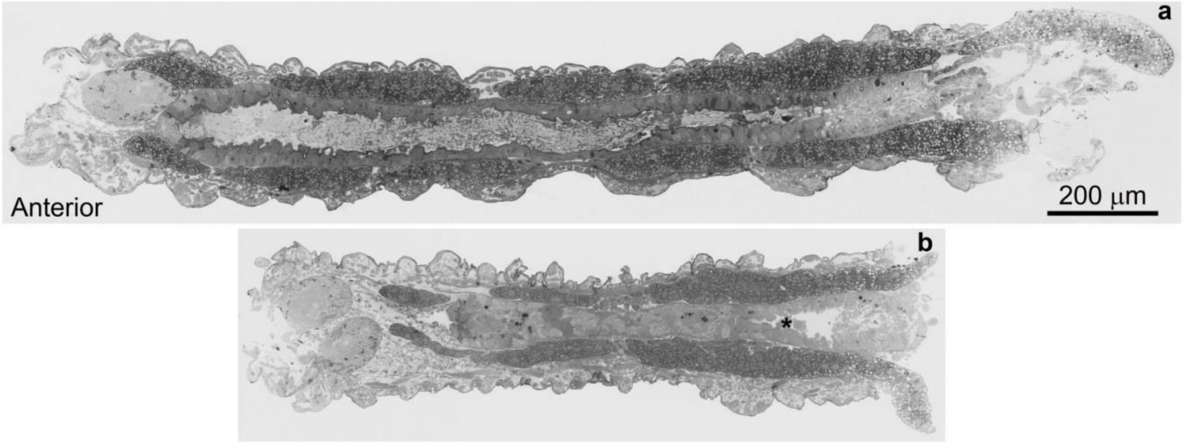

Supplementary Figure 4. Longitudinal cross sections of nearly entire neonates untreated (**a**) and treated (**b**) with *dvssj1* dsRNA, as seen in the scanning electron microscope via backscattered electron imaging at the same magnifications. Note the significantly reduced size and occlusion of the gut lumen of the treated individual (b) relative to that of the control. Evidence of highly active epithelial regeneration in the treated neonate is manifest as numerous enterocytes blebbing into the posterior midgut lumen (*). Neonate anteriors are positioned to the left in both cases.

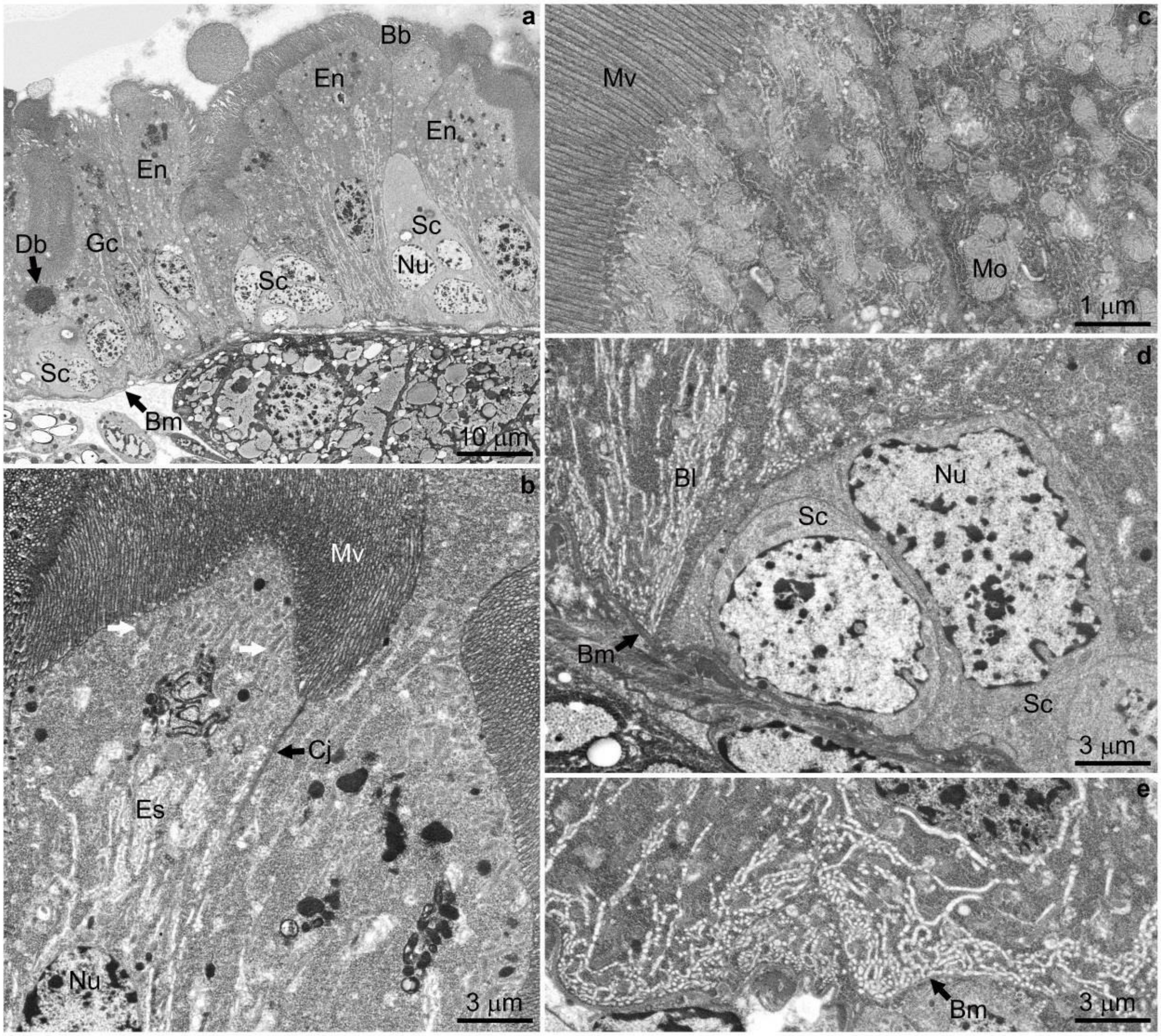

Supplementary Figure 5. Anterior midgut epithelium from untreated neonates.

(a) Region of midgut epithelium undergoing regeneration as evidenced by enlarged stem cells (Sc), the presence of a goblet cell (Gc) "dark body" (Db) apparently consisting of nascent microvilli, and blebbing of enterocytes into the gut lumen (top). Note the numerous electron dense cytoplasmic vesicles/tubules in the sub-brush border region of cells, and the prominent basal extracellular labyrinth that extends from the basal membrane of enterocytes upwards well beyond nuclei. Bb, brush border; Bm, basal membrane; En, enterocyte; Nu**,** nucleus. **(b)** Apical region of two epithelial cells. The sub-apical cytoplasm includes large numbers of mitochondria (white arrows), copious endoplasmic reticulum and free ribosomes, as well as electron dense vesicles/tubules and endosomal compartments (Es). Cj, cell junction; Mv, microvilli. **(c)** Cytoplasm adjacent to the apical membrane is densely populated with mitochondria (Mo) and endoplasmic reticulum (non-osmicated specimen). **(d)** Note prominent basal extracellular labyrinth (Bl) extending from the basal membrane (Bm), and the absence of any dark inclusion bodies from the cytoplasm of stem cells (Sc). **(e)** Typical basal region of enterocytes of untreated neonate anterior midgut exhibiting basal extracellular labyrinth, mitochondria and endoplasmic reticulum.

a.

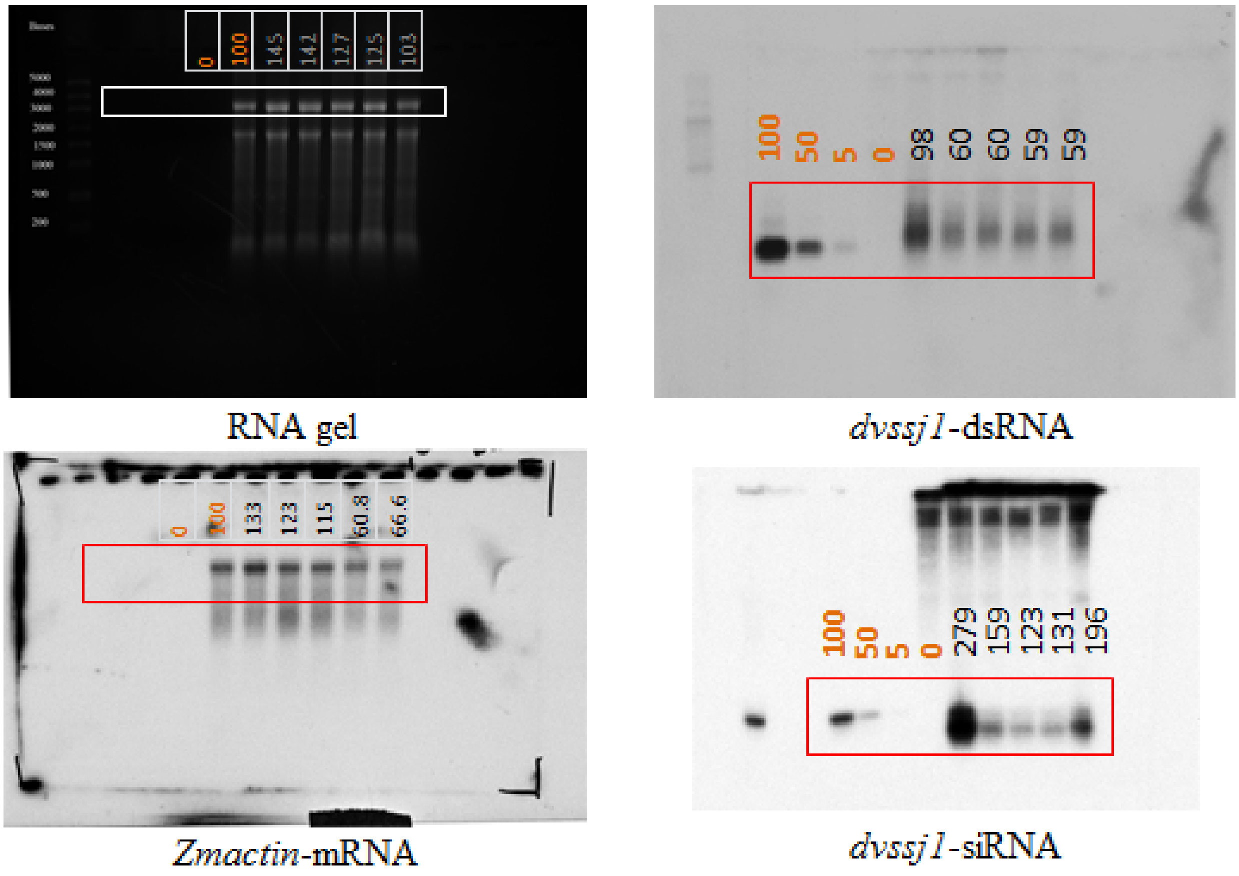

b.

gctaactaactagg **ataataagttcgatttttacgaaaatgacaagtatcgagactgtggggaccat**
tgtcctgaaattgctgaagttggtgatcaatttgatatgtctcatcttgtaccgaaccggatatcaagg
ctacttcttgggagtaggaggaacctggaatctaaacgaagaaaaaatcccgatgcagaaattgtggc
ttccggcgtattcgtagg taattgagaattcgatatcaggtccgccttgtttctcctctgtctcttga
tctgactaatcttggtttatgatcttgatgatttagcttgactatgcgattgctttcctggacccgtgc
agctgcccatcgaccctcaatta **cctacgaatacgccggaagccacaatttctgcatcgggattttt**
tcttcgtttagattccaggttcctcctactcccaagaagtagccttgatatccggttcggtacaagatg
agacatatcaaattgatcaccaacttcagcaatttcaggacaatggtccccacagtctcgatacttgtc
attttcgtaaaaatcgaacttattat cctagttagttagg

Supplementary Figure 6. Original northern blots of five *dvssj11* events and one transgenic negative isoline (red boxes in Figure 7).
(a) Double-strand *dvssj11* frag1 (210 bp, 100, 50 5 pg) RNA was loaded as positive control and *Zmactin* (Accession #: EU952376) was included as a reference gene for northern analysis to re-probe same blot; 29nt *dvssj11* oligo (100, 50 5 pg) was used as a positive control for the siRNA northern blot. Densitometry analyses of northern blots were carried out with Phoretixs 1D software (Cleaver Scientific, UK) and relative amount (pg) of each lane was presented (Supplementary Table 3). (**b**) Sequences of *dvssj1* hairpin design. When single 5' and 3' untranslated region and loop region were removed, 210 bp of *dvssj11* frag1 (bold) was flanked with 14 bp and 8 bp to form 232 bp dsRNA transcripts (underline).

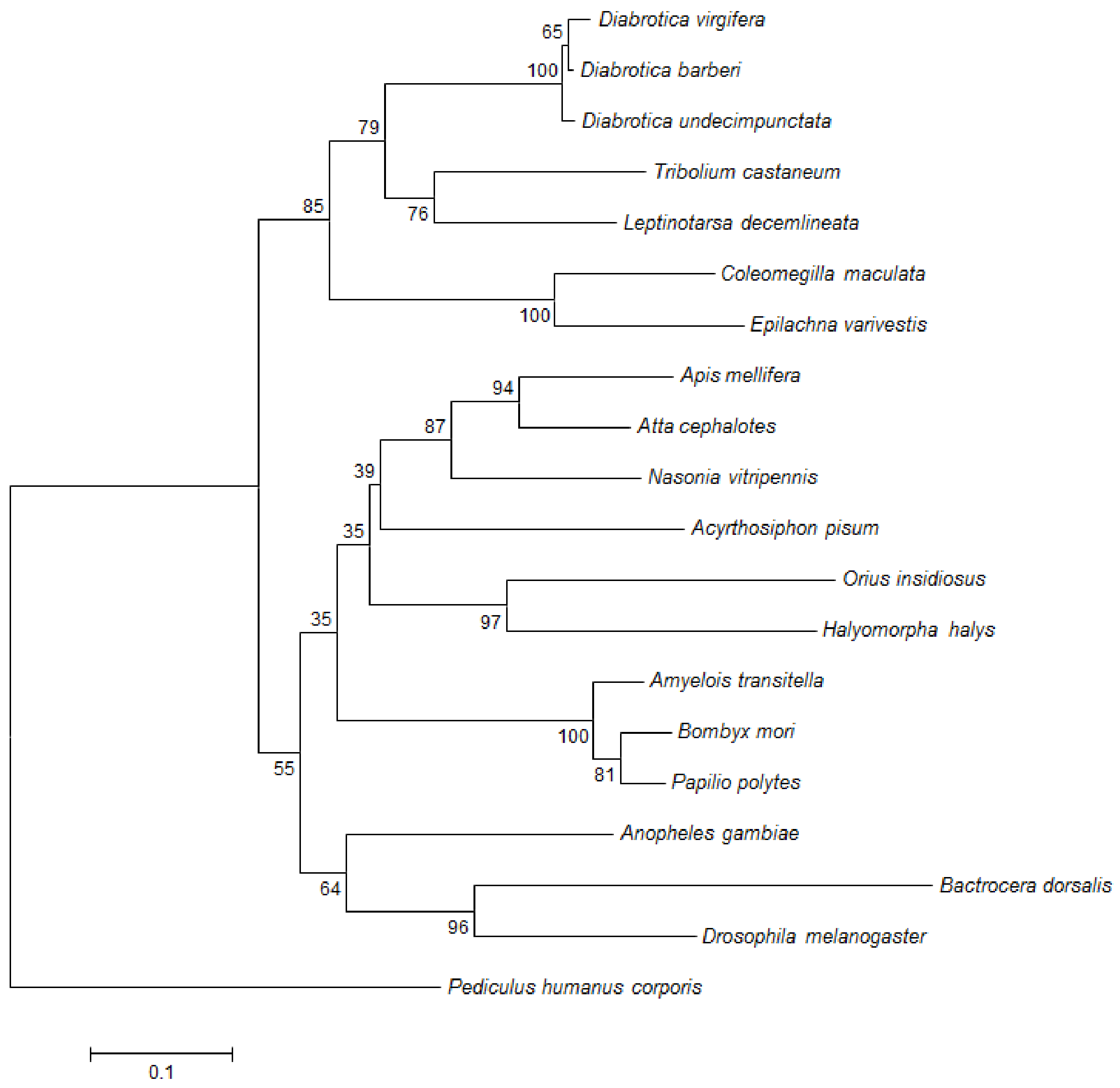

Diabrotica virgifera
Diabrotica barberi
Diabrotica undecimpunctata
Tribolium castaneum
Leptinotarsa decemlineata
Coleomegilla maculata
Epilachna varivestis
Apis mellifera
Atta cephalotes
Nasonia vitripennis
Acyrthosiphon pisum
Orius insidiosus
Halyomorpha halys
Amyelois transitella
Bombyx mori
Papilio polytes
Anopheles gambiae
Bactrocera dorsalis
Drosophila melanogaster
Pediculus humanus corporis
65
100
79
76
85
100
94
87
39
35
97
35
100
81
55
64
96
0.1

Supplementary Fig 7. Evolutionary relationships of selected orthologs of DVSSJ1 representing different insect orders.

The evolutionary history was inferred using the Neighbor-Joining method[1]. The optimal tree with the sum of branch length = 3.773 is shown. The percentage of replicate trees in which the associated taxa clustered together in the bootstrap test (1000 replicates) are shown next to the branches[2]. The tree is drawn to scale, with branch lengths in the same units as those of the evolutionary distances used to infer the phylogenetic tree. The evolutionary distances were computed using the Poisson correction method[3] and are in the units of the number of amino acid substitutions per site. The analysis involved 20 amino acid sequences. All ambiguous positions were removed for each sequence pair. There were a total of 177 positions in the final dataset. Evolutionary analyses were conducted in MEGA7[4]. SSJ proteins of following selected homologs of DVSSJ1 were analyzed this tree**:** *Diabrotica virgifera* (Seq.No. 693), *Diabrotica barberi* (Seq.No. 695), *Diabrotica undecimpunctata* (Seq.No.694), *Leptinotarsa decemlineata* (Seq.No. 697), *Epilachna varivestis* (Seq.No. 696), *Coleomegilla maculate* (Seq.No. 699), *Orius insidiosus* (Seq.No. 698), *Tribolium castaneum* (XP_008197065.1), *Anopheles gambiae* (XP_311473.3), *Bactrocera dorsalis* (XP_011205009.1), *Drosophila melanogaster* (NP_649184.1), *Acyrthosiphon pisum* (NP_001156315.1), *Halyomorpha halys* (XP_014277667.1), *Apis mellifera* (XP_003249659.1), *Atta cephalotes* (XP_012058457.1), *Nasonia vitripennis*, (XP_003424573.1), *Amyelois transitella* (XP_013199749.1), *Bombyx mori* (AK378208.1), *Papilio polytes* (XP_013143312.1), and *Pediculus humanus corporis* (XP_002427111.1). Seq. Nos were disclosed in US20140275208 A1.

Supplementary Table 1a. Identification of dsRNA active targets against WCR

cDNA ID	dsRNA size	primary Score	confirm-ation	Tc orthologs[a]	E-value	% identity	Annotation[d]	CG orthologs[b]	Seq ID[c]
idv1c.pk037.j20.f	593	3.0	3.0	TC008407	4.50E-68	61	Protein mesh-like Protein; named *dvssj2*	mesh/CG31004	Seq No.001
idv1c.pk034.k22.f	445	2.6	2.0	TC013063	2.20E-60	98.0	DNA directed RNA polymerase II 18kD subunit	RpII18/CG1163	Seq No.009
iwc1c.pk019.o21	204	2.1	3.0	TC009191	8.20E-19	67.2	Protein transport protein Sec23A-like Protein	Sec23/CG1250	Seq No.017
iwc1c.pk026.h16	635	1.8	2.4	TC001073	2.40E-109	86.4	T-complex protein 1 subunit alpha-like Protein	CG8258	Seq No.037
iwc1c.pk029.h21	670	2.4	2.3	TC008784	1.60E-22	84.4	Elongation factor 2-like Protein	EF2/CG2238	Seq No.045
iwm2c.pk004.m2	528	2.6	2.6	TC002574	3.20E-23	82.9	Signal recognition particle 54 kDa protein-like Protein	Srp54k/CG4659	Seq No.049
iwm2c.pk005.l7	583	2.3	1.8	TC007744	6.20E-112	91	T-complex protein 1 subunit gamma-like Protein	Cct5/CG8439	Seq No.057
iwm2c.pk008.e24	588	2.5	1.9	TC012303	3.40E-111	98.3	Eukaryotic translation initiation factor 3 subunit A	eIF3-S10/CG9805	Seq No.061
idv1c.pk015.b8.f	716	3.0	3.0	TC011289	2.70E-77	74.7	GTP-binding protein SAR2	Sar1/CG7073	Seq No.065
idv1c.pk019.h19.f	619	2.1	2.4	TC013376	1.60E-55	78.8	Homocysteine S-methyltransferase 2-like Protein	CG10623	Seq No.069
idv1c.pk026.d10.f	319	2.6	3.0	TC002583	3.30E-56	98.9	Histone H2A	His2A/CG31618	Seq No.077
idv1c.pk037.j14.f	587	2.6	2.3	TC009158	6.30E-127	98.4	Ras-like GTP-binding protein Rho1	Rho1/CG8416	Seq No.085
idv1c.pk038.d14.f	614	2.6	2.9	TC014413	2.00E-91	93.9	ATP-dependent RNA helicase WM6-like Protein	Hel25E/CG7269	Seq No.101
idv1c.pk040.c10.f	368	2.6	2.0	TC002461	1.70E-48	74.5	Nuclear transport factor 2 (NTF2) domain protein	Nxt1/CG12752	Seq No.113
idv1c.pk042.g10.f	628	2.0	1.4	TC002738	6.30E-106	89.9	Eukaryotic translation initiation factor 3 subunit E	Int6/CG9677	Seq No.125
idv1c.pk042.i20.f	619	2.1	2.1	TC001437	1.60E-113	96.0	AP-1 complex subunit mu-1-like Protein	AP-1mu/CG9388	Seq No.129
idv1c.pk002.j17.f	589	2.4	2.5	TC008015	2.10E-96	81.7	Proteasome subunit alpha type	Prosalpha7/CG1519	Seq No.137
idv1c.pk003.d6.f	577	2.3	1.8	TC000069	2.50E-109	83.3	Proteasome subunit beta type-1-like Protein	Prosbeta6/CG4097	Seq No.141
idv1c.pk016.h19.f	542	2.6	2.1	TC008665	1.10E-85	89.1	Proteasome subunit beta type	Prosbeta1/CG8392	Seq No.145
idv1c.pk025.a4.f	559	2.6	2.6	TC014286	1.60E-116	94.6	Proteasome non-ATPase regulatory subunit 3-like protein	Rpn3/CG42641	Seq No.149
idv1c.pk047.d23.f	529	2.4	2.0	TC000258	8.20E-102	91.2	Proteasome subunit alpha type	Prosalpha6/CG4904	Seq No.165
idv1c.pk045.e5.f	628	2.3	2.3	TC003184	2.60E-24	88.7	Dosage compensation regulator-like Protein	mle/CG11680	Seq No.237
idv1c.pk046.p17.f	524	2.4	2.6	TC014786	4.10E-63	94.1	small GTP binding protein RAB5	Rab5/CG3664	Seq No.245
idv1c.pk054.k12.f	546	2.1	2.0	TC007406	2.60E-92	89.4	60S ribosomal protein L9-like Protein	RpL9/CG6141	Seq No.301
idv1c.pk057.h7.f	670	2.4	2.4	TC009126	3.80E-112	83.9	Heat shock protein 68a	Hsc70-4/CG4264	Seq No.309
idv1c.pk057.n10.f	672	2.0	2.4	TC012133	7.70E-112	95.2	V-type proton ATPase subunit B	Vha55/CG17369	Seq No.313
idv1c.pk058.b17.f	500	2.3	2.1	TC001251	9.70E-78	100	Calmodulin-like Protein	CG17770	Seq No.317
idv1c.pk055.m8.f	718	2.3	2.3	TC009965	1.10E-98	98.7	Ras-related protein Rab-3-like Protein	Rab9Fb/CG32670	Seq No.329
idv3c.pk007.i8.f	440	2.3	2.1	TC006296	6.10E-81	98.4	Actin-5C-like Protein	Act5C/CG4027	Seq No.353
idv3c.pk008.h22.f	600	2.1	1.9	TC011025	6.20E-66	98.5	V-type proton ATPase proteolipid subunit	Vha16-5/CG6737	Seq No.357
idv3c.pk012.e23.f	551	2.0	1.8	TC004721	9.70E-59	88.8	Twitchin-like Protein	bt/CG32019	Seq No.365
idv3c.pk013.g12.f	586	2.0	2.0	TC001225	2.80E-78	98.9	Polyubiquitin	Ubi-p63E/CG11624	Seq No.369
idv3c.pk016.a10.f	611	2.1	2.0	TC001574	3.60E-65	73.7	actin-depolymerizing factor 1 [Bombyx mori]	CG6873	Seq No.373
idv1c.pk035.i17.f	1156	2.8	n/a	TC005653	4.80E-59	82.1	Putative uncharacterized protein; named *dvssj1*	Ssk/CG6981	Seq No.573 or 234*
idv1c.pk001.e9.f	566	1.57	2	TC007928	8.60E-74	90.4	40S ribosomal protein S10b-like Protein	RpS10b/CG14206	Seq No. 8*

[a]Top TC orthologs were identified through TBLASTX against *Tribolium castaneum* Tcas3 (Genomic sequence); [b]Top CG orthologs were identified through TBLASTX against *Drosophila melanogaster* BDGP6 (Genomic sequence); [c]Sequences of RNAi active WCR genes were disclosed in US20110054007A1* and US 20140275208A1; [d]Annotation of top TC or CG hits was made *via* UniProtKB/Swiss-Prot

Supplementary Table 1b. PCR primers for clone-specific amplification of WCR cDNAs to produce dsRNA for diet-based RNAi primary and confirmation screening

cDNA ID	dsRNA size	Seq ID[c]	PCR forward primer	PCR reverse primer
idv1c.pk037.j20.f	593	Seq No.001	TGACTTCACACTTCCAGATGGT	TCGTTGAGAGTATTCCTGCTGA
idv1c.pk034.k22.f	445	Seq No.009	GTTTCGTTTCATCATCATTT	TCGTCTATTCCCCAATCTTCAT
iwc1c.pk019.o21	204	Seq No.017	CCCAATGCCGAGATATATTG	AGGTATTATGCTGTGGACGA
iwc1c.pk026.h16	635	Seq No.037	AATGGCTTTACACGTCCCTAAA	AACCAGACCCCAAAACTTTACA
iwc1c.pk029.h21	670	Seq No.045	TCGTAATTTTTGGTTGTGCTTG	TCAACTCTGACTTCACCCATTG
iwm2c.pk004.m2	528	Seq No.049	CGTCAAAAGTATTTGTCGCATC	TTTTACCTGATCATAGGCACCA
iwm2c.pk005.l7	583	Seq No.057	GCACGAGGCTTTATTAGACAGAG	TAAGTGATTGGCTTCATCATCG
iwm2c.pk008.e24	588	Seq No.061	AGAGAACAGTCAGCACAAGCAG	GAAGAACTTGAACAAAGCAGCA
idv1c.pk015.b8.f	716	Seq No.065	TTGAAAAAGATAGGGCTCCAAA	CAATGACAGAACGAAATGGAAA
idv1c.pk019.h19.f	619	Seq No.069	TCGGTCGATTTAGGTAGTAGGTG	GTAGTTTTCGCATAGGCTCCTG
idv1c.pk026.d10.f	319	Seq No.077	TGGTAAGGGAGGCAAAGTTAAG	ATTAGGCAATACTCCACCTTGG
idv1c.pk037.j14.f	587	Seq No.085	AACTTTGTGTGTTTTGGCAGGT	GGGGCTTCTTCTTCTTTTTG
idv1c.pk038.d14.f	614	Seq No.101	CAACAATTTTCGCATCTTTCTG	TTTTTCGATCTGACCAATGCTA
idv1c.pk040.c10.f	368	Seq No.113	GTTGCAGAAGATTTCACAAAA	TAGCGTTTAATGGTTCTTGCAG
idv1c.pk042.g10.f	628	Seq No.125	CCAGTATTTAGACCGGCATCTC	ATGGATCAACCAAGTCCTCTGT
idv1c.pk042.i20.f	619	Seq No.129	TCACATTCCAGTTGTTTAGA	TCTTAGTACATTGCCATTTG
idv1c.pk002.j17.f	589	Seq No.137	CGATTTATCAGCTTCCCAATTC	CAGCTCATCATGGACCAAATAA
idv1c.pk003.d6.f	577	Seq No.141	AATTGGTGCAGATACACGTTTG	CTAATCCCGTCTCAACTGGAAG
idv1c.pk016.h19.f	542	Seq No.145	TCTTCGTTCAATACCAGCTTCA	AGTTTGATGGAGGCGTTGTTAT
idv1c.pk025.a4.f	559	Seq No.149	CTGAATTGACCAACAAATTGGA	TTAATAACATTGTGGCGCAGTC
idv1c.pk047.d23.f	529	Seq No.165	ATTTTTGAGGTTGTCGGCTAGA	AGAAGGGCACGTTTGGTAGATA
idv1c.pk045.e5.f	628	Seq No.237	TCATTGTGTCCTAAAGGCATGA	AAAATCACCGACTACGTCATCA
idv1c.pk046.p17.f	524	Seq No.245	GGTTGCCGTCGTTTAGTAGAAG	CCGAATGTGTCTTGATTGGTTA
idv1c.pk054.k12.f	546	Seq No.301	CAAGTAGGGCTAAAATGAAGCAA	AAAGCCCATCTAAGAATTTACGG
idv1c.pk057.h7.f	670	Seq No.309	ATTGCAGTCATTGCTGTAGTGC	CGAAAATGAGGACGTTTCTTTC
idv1c.pk057.n10.f	672	Seq No.313	TACACGTAAAAATGGCTTCGAC	TTAACACCCATAGCAGCGAAC
idv1c.pk058.b17.f	500	Seq No.317	ACTTTGACGAGAGGCGTTGT	CGTAATTGACTTGACCATCACC
idv1c.pk055.m8.f	718	Seq No.329	GTTGGGATTGGGATTTAACAAA	TTGATCGAACCTAACCTTATTTCC
idv3c.pk007.i8.f	440	Seq No.353	GTAATGAAAGATTCCGTTGT	CCCAAGATGGGGGATATAAAA
idv3c.pk008.h22.f	600	Seq No.357	GTTCGCTAGTTCCGTAGTGTGTT	CAACTTTTGGTACGCTGTGTTT
idv3c.pk012.e23.f	551	Seq No.365	GATCTTTAGTCAGTGCTTT	TGGGGCTGTAGTTGAGATTGA
idv3c.pk013.g12.f	586	Seq No.369	GAGTAGTCGGTATGTATTT	ATTTCTCATCTCCTGGTTTGGA
idv3c.pk016.a10.f	611	Seq No.373	CTCCGAAGACAGACAGTCGAT	AAATAATACACCTCGGCAGGTC
idv1c.pk035.i17.f	1156	Seq No.573	ATAATAAGTTCGATTTTTTACG	GCAGACAAAACCCATCAAGCATG
idv1c.pk001.e9.f	566	Seq No. 8*	CTTTCTGATTTTTGACAG	AACCAAACTTTTTATTGGCTGATC

Supplementary Table 2. WCR feeding results of additional active targets and fragments

dsRNA Name	base pair	primary score	LC50 ng µl-1	IC50 ng µl-1	Forward primer	Reverse primer
*dvssj1*FIS	1156	2.8	n/a	n/a	GGAGATCACAAAACGACATTCA	TATGAAGAGAACGGGAGGAAAA
***dvssj1* frag1**	210	2.9	0.045	0.014	ATAATAAGTTCGATTTTTTACGAAAATG	TACGAATACGCCGGAAGC
*dvssj1*frag2	145	3	0.098	0.014	ACGAAAATGACAAGTATCGAGA	CTTCGTTTAGATTCCAGGTTC
dvssj1 frag5	502	2	0.163	0.3	ATAATAAGTTCGATTTTTTACGAAAATG	TTGCATTTTGGCTTTGAGGATAAAG
dvssj2 FIS	573	3	1.738	0.366	TGACTTCACACTTCCAGATGGT	TCGTTGAGAGTATTCCTGCTGA
dvssj2 frag1	225	2.6	0.447	0.149	CGACAAGGAGGAAAGTTGTG	TCGTTGAGAGTATTCCTGCTG
***dvssj2* frag7**	162	2.4	0.121	0.054	TTTTGTTTTGTTTAGTGTTAGCGTTAAAGG	TGGAGGGACTCCATTAGGCGTAT
dvrps10 FIS	600	1.57	n/a	n/a	GGGGCTTTCTGATTTTTGACAG	AACCAAACTTTTTATTGGCTGATC
dvrps10 frag1	263	1.75	200	2.81	GACTAACTCTGGCATCGAATACCTC	GTGGTGCCCGTCCACGTC
dvrps10 frag2	208	1.88	131	3.4	GTCCCATCTACCTTGAAACGC	GTGGTGCCCGTCCACGTC
***dvrps10* frag4**	557	2.88	0.146	0.03	AAGGTTACGTAAAGGAACAA	GAGTCCTTCTGTATCCTGCA
dvpat3 FIS	1043	1.13	n/a	n/a	GGGGAGTCGTCAACATCAATTTC	GACATAAAGTTATGTATTTATTAGAAAAAAAAG
dvpat3 frag5	322	2.5	27.98	0.992	GGAGTCGTCAACATCAATTTCA	GCCTATTACGGTGCCACTATTT
dvpat3 frag8	161	2.5	1.995	0.223	CAAGTTGAATATGCAATGAAAGCAG	CCTATGTGTTTATCAATGTTGAAAATTCG
dvpat3 frag10	161	2.5	88.86	0.38	GACCATTTGGTTGCAGTGTCATCTTG	CCCCATCTTCAATTTTTCTATTTCAG
***dvpat3* frag13**	161	2	0.54	0.023	AATATGGTTCAAATATTCCTCTTAAATAC	TCCAGATGGATCAATCAGATACATAG
dvprtb FIS	913	2.5	4.105	0.186	GGGGGCAGTTATTTCGACTTTTC	GTTTATTTAGAAAGTATATTTGAATTACACATAAG
***dvprotb* frag1**	203	2.13	4.558	0.047	TACCAGAGAACAAAACAAACTTTTCC	AAGGAAAGAATCTCTTGTAGTAGAGC

The most active fragments of five RNAi active targets were selected (bold) for LT_{50} comparison (Table 2). *Dvpat3* frag13 is a 161 bp sequence from Proteosome alpha 3 type subunit (*pat3*); *dvprotb* frag1 is a 203 bp sequence from Proteosome subunit beta type 1 (*protb*), and *dvrps10* frag4 is a 557 bp sequence from the ribosomal protein s10e gene.

Supplementary Table 3. Primer and probe designs for qRT-PCR, qPCR, in situ hybridization (ISH) and northern analyses

**Designation**[a]	C°	**Sequence or source**
**qRT-PCR**[b]		**primer designs for WCR expression study**
WCR *dvssj1* F	60	CGTGAAGCCCAGTAAATACCT
WCR *dvssj1* R		GTTTTGGTTTTATTTTAATTGCAGACAA
WCR *dvssj1* P (FAM)		AAACCCATCAAGCATGGCAGTTCC
WCR *dvrps10* F	60	GATGTTGAGTTCAGGCAAGGATT
WCR *dvrps10* R		CCAAACTTTTTATTGGCTGATCAAT
WCR *dvrps10* (VIC)		ACGTGGACGGGCAC
**qPCR**		**primer designs for transgenic copy number**[c]
dvssj1-frag1 F	60	CTTGACTATGCGATTGCTTTCCT
dvssj1-frag1 R		TCCGGCGTATTCGTAGGTAATT
dvssj1-frag1 P		CTGCCCATCGACCCT
NLB F	60	TGATTCCGATGACTTCGTAGGTT
NLB R		GCTAATCGTAAGTGACGCTTGGA
NLB P		TAGCTCAAGCCGCTCG
NRB F	60	CATGAAGCGCTCACGGTTACTAT
NRB R		TCGTACGCTACTGCCACCAA
NRB P		ACGGTTAGCTTCACGACT
***In situ* hybridization**		**RNA probes**[d]
dapB probe		SKU: 310043; RNAscope® Probe as negative control probe
dvssj1 RNA probe (357 nt); *dvssj1* (KU562965) from 105 to 461 of orf		CUUGGGAGUAGGAGGAACCUGGAAUCUAAACGAAGAAAAAAAUCCCGAUGCAGAAAUUGUG<br>GCUUCCGGCGUAUUCGUAGGAUUUAUGAUUUACACAUUCGUCUCGCUGAUCAGCCUUUGCU<br>UCGCUAGUGGAGAUCACAAAACGACAUUCACUGAUAUUCUGAUGAAUAUAGUAGGGAUUUU<br>UAUGUGGAUAGCUGUUGGAGCUACAGCUCUUCAUUAUUGGCUUGGGUACUUGUCCGAAUA<br>CAAAUACACGACAAUAGAUUCUGAACGACAAGUUGGUUUGGCGUUAGGAGCGAUGUGUAUA<br>AUAAAUGGAGCGGUCUAUCUUGUAGACGGAGUACUUUCC
**Northern analysis**		**probe sequences or source**
dvssj1-frag1	65	ATAATAAGTTCGATTTTTTACGAAAATGACAAGTATCGAGACTGTGGGGACCATTGTCCTG<br>AAATTGCTGAAGTTGGTGATCAATTTGATATGTCTCATCTTGTACCGAACCGGATATCAAG<br>GCTACTTCTTGGGAGTAGGAGGAACCTGGAATCTAAACGAAGAAAAAAATCCCGATGCAGA<br>AATTGTGGCTTCCGGCGTATTCGTAGG
zmactin	65	Accession #: EU952376
dvssj1 29 nt oligo control	37	GATAATAAGTTCGATTTTTTACGAAAATG

[a]Sequences of PCR primers (F=forward; R=reverse) and probes (P) were listed; [b]qRT-PCR was conducted on Quant Studio 7Flex for 35 cycles; [c]qPCR was performed on Roche LightCycler® 480 for 40 cycles; [d]Sequences of target probes, preamplifier, amplifier, and label probe are designed by Advanced Cell Diagnostics (Hayward, CA); [o]Temperatures of PCR annealing or hybridizations; NRB: A synthetic sequence designed to facilitate PCR analysis of transgene insert near right border (RB) of T-DNA; NLB: A synthetic sequence designed to facilitate PCR analysis of transgene insert near left border (LB) of T-DNA.

Supplementary Table 4. Characterization of T1 transgenic dvssj1 plants

Event	NRB copy #	*dvssj1* copy #	NLB copy #	Number of plants	Mean Score	Differen ce	Std Err Diff	p-Value	dsRNA band (pg)	siRNA band (pg)
6.1.25	3	4	3	14	0.1179	1.3655	0.1307	<.0001*	98	279
6.4.2	0	1	2	14	0.1214	1.3987	0.1333	<.0001*	60	159
6.4.18	1	1	1	15	0.1667	1.3167	0.1284	<.0001*	60	123
6.2.7	1	1	1	15	0.4000	1.0833	0.1284	<.0001*	59	131
6.2.3	0	1	1	15	0.6100	0.8733	0.1284	<.0001*	59	196
NULL	0	0	0	15	1.4833	-	-	-	0	0

T1 transgenic seeds were planted and leaf samples were used for qPCR analysis[5] of copy number of transgene, which detected *dvssj1* hairpin region and sequences near LB (NLB) or RB (NRB) of T-DNA (Supplementary Table 3). Only PCR positive plants were selected for WCR bioassay and northern blot analyses. Statistical calculations were performed using JMP 12.0.1 statistical software. Data were checked for normal distribution by Shapiro-Wilk W Test ($p<0.0001$) indicating that the data does not follow a normal distribution. Extreme outliers were identified and excluded using Grubb's Outlier Test. To assess the root protection from corn rootworm feeding conveyed by *dvssj1* in transgenic plants the data were analyzed by non-parametric one-way ANOVA (Kruskall-Wallis) showing there was a difference detected among events ($p<0.0001$). Dunnett's *post hoc* test was used to compare all treatments against the control treatment, NULL. There is significant evidence identifying a difference in nodal injury score between the NULL and all other events, each with a p-value <0.0001. Densitometry analyses of northern blots were carried out with Phoretixs 1D software and relative amount (pg) of each event based on positive control (100 pg) in Supplementary Fig 7.

Supplementary Table 5. List of DVSSJ1 orthologs.

**Scentific name**	**Common name**	**Order**	**Seq No./Acession #**
Diabrotica virgifera	western corn rootworm	Coleoptera	Seq. No.693
Diabrotica barberi	northern corn rootworm	Coleoptera	Seq. No.695
Diabrotica undecimpunctata	southern corn rootworm	Coleoptera	Seq. No.694
Leptinotarsa decemlineata	Colorado potato beetle	Coleoptera	Seq. No.697
Epilachna varivestis	Mexican bean beetle	Coleoptera	Seq. No.696
Coleomegilla maculata	spotted lady beetle	Coleoptera	Seq. No.699
Tribolium castaneum	red flour beetle	Coleoptera	XP_008197065.1
Anopheles gambiae	mosquitoes	Diptera	XP_311473.3
Bactrocera dorsalis	oriental fruit fly	Diptera	XP_011205009.1
Drosophila melanogaster	fruit fly	Diptera	NP_649184.1
Acyrthosiphon pisum	pea aphid	Hemiptera	NP_001156315.1
Halyomorpha halys	brown marmorated stink bug	Hemiptera	XP_014277667.1
Orius insidiosus	insidious flower bug	Hemiptera	Seq. No.698
Apis mellifera	honey bee	Hymenoptera	XP_003249659.1
Atta cephalotes	leafcutter ant	Hymenoptera	XP_012058457.1
Nasonia vitripennis	jewel wasp	Hymenoptera	XP_003424573.1
Amyelois transitella	navel orangeworm	Lepidoptera	XP_013199749.1
Bombyx mori	silkworm	Lepidoptera	AK378208.1
Papilio polytes	common Mormon	Lepidoptera	XP_013143312.1
Pediculus humanus corporis	human body louse	Phthiraptera	XP_002427111.1

*Dvssj1*orf was used to search Ensembl genomes and NCBI database (tBlastx; E-value threshold=1e-1; BLOSUM 62 or BOLSUM 45) and no hits was found from human, mouse, maize, *C. elegans* or other organisms outside of arthropod. Only selected sequences representing different groups of insects were listed in this table and built for consensus tree (Supplementary Fig. 6). Seq. Nos were insect transcripts disclosed in US20140275208 A1.

Supplementary Method

Microscopy

Neonates were collected after 72 h incubation with dsRNA (100 ng μl^{-1}) and prepared for electron microscopy as follows. An anesthesia chamber was prepared by placing an aluminum pan filled with CO_2 pellets in the bottom half of a vacuum desiccator (Bel-Art, Wayne, NJ), and covered with the desiccator's lid with the air valve open. Only neonates having consumed the amended diet as determined by direct observation of Cy3 fluorescence signal in the digestive tract were selected; non-feeders were discarded. Petri plates with selected neonates were moved to an anesthesia chamber and placed on a Styrofoam block on a shelf above CO_2 pellets for 20 minutes. Neonates were fixed in a solution of 5% acrolein (Sigma, St. Louis, MO) and 0.25% glutaraldehyde (Electron Microscopy Sciences, EMS, Hatfield, PA) in 100 mM cacodylate buffer pH 7.2, with 1 mM $CaCl_2$ and 100 mM sucrose. Anesthetized neonates were rapidly transferred to the filter paper saturated with fixation solution and additional fixation solution added until they were nearly submerged for an initial fixation of 30 minutes. With the aid of a dissecting microscope, an ophthalmic scalpel (EMS, Hatfield, PA) was used to remove the proximal and distal ends of each neonate to allow further penetration of the fixative and embedding resin, but without cutting through the digestive tract. Neonates were transferred to a 20 ml glass scintillation vial containing 10 ml of fresh fixation solution on a rotisserie at room temperature for 4 h, and then to a rotisserie at 4°C overnight. Neonates were rinsed 3 x 30 minutes in cacodylate buffer (minus the sucrose) on a rotisserie at room temperature. Some neonates were then post-fixed in 1% OsO_4 in cacodylate buffer for 3 h at room temperature, then at 4°C overnight. All micrographs depict osmicated specimens unless noted otherwise. Samples were rinsed on a rotisserie at room temperature 3 x 30 minutes in cacodylate buffer, then in ddH_2O, and transferred

to 5% ethanol in ddH_2O overnight. Dehydration continued using 20%, 50%, 80% ethanol, and three rinses in 100% ethanol, one hour on a rotisserie at room temperature for each change. Resin embedding was achieved by infiltration with LR White (Sigma, St. Louis, MO) in ethanol at 5%, 20%, 50%, 80%, and three changes at 100%, 24 h between changes, on a rotisserie at room temperature. Infiltrated neonates were placed into gelatin capsules (Ted Pella, Redding, CA) which were then filled with fresh LR White, capped, and polymerized at 60°C for 72 h. We adapted the methods described elsewhere for subsequent preparation and examination of ultrastructure via backscattered electron imaging in a scanning electron microscope

Supplementary References:

1 Saitou, N. & Nei, M. The neighbor-joining method: a new method for reconstructing phylogenetic trees. *Mol. Biol. Evol.* **4**, 406-425 (1987).

2 Felsenstein, J. Confidence Limits on Phylogenies: An Approach Using the Bootstrap. *Evolution* **39**, 783-791, doi:10.2307/2408678 (1985).

3 Zuckerkandl, E. & Pauling, L. in *Evolving Genes and Proteins.* pp.97-166 (Academic Press, 1965).

4 Kumar, S., Stecher, G. & Tamura, K. MEGA7: Molecular Evolutionary Genetics Analysis version 7.0 for bigger datasets. *Molecular Biology and Evolution*, doi:10.1093/molbev/msw054 (2016).

5 Zhi, L. *et al.* Effect of Agrobacterium strain and plasmid copy number on transformation frequency, event quality and usable event quality in an elite maize cultivar. *Plant Cell Rep.* **34**, 745-754, doi:10.1007/s00299-014-1734-0 (2015).
